# Targeting the oxidative stress-neuroinflammation axis: the mechanism of arctigenin’s broad-spectrum analgesia with limited side effects

**DOI:** 10.3389/fimmu.2026.1754756

**Published:** 2026-03-05

**Authors:** Zhe Wang, Shu Li, Ping Lu, Jinglei Liao, Yimin Xu, Chen Lu, Weiwei Li, Jinhong Jiang

**Affiliations:** 1 School of Food and Biological Engineering, Xuzhou University of Technology, Xuzhou, China; 2 Jiangsu Province Key Laboratory of Anesthesiology and Brain Science, Jiangsu Province Key Laboratory of Anesthesiology, Xuzhou Medical University, Xuzhou, Jiangsu, China; 3 Pharmacy College of Shihezi University/Key Laboratory of Xinjiang Phytomedicine Resource and Utilization, Ministry of Education/Collaborative Innovation Center for Efficient Safflower Production and Resource Utilization of XPCC/Institute for Safflower Industry Research, Shihezi University, Shihezi, China; 4 Department of Anesthesiology, Xuzhou Maternal and Children Health Care Hospital, Xuzhou, China

**Keywords:** arctigenin (AG), c-fos, MAPK, microglia, mitochondrial, neuropathic pain

## Abstract

**Background:**

Arctigenin (AG), a natural lignan compound, has been reported to reveal its anti-inflammatory effects in glucose, lipid metabolism and type 2 diabetes mellitus. An increasing number of studies suggest that microglia activation evoked neuroinflammation is known to contribute to the development and progression of neuropathic pain. This study aims to investigate the role and mechanism of AG in ameliorating spared nerve injury (SNI)-induced neuropathic pain.

**Materials and methods:**

SNI model was defined as suffering severe hyperalgesia and allodynia and established in C57BL/6 male mice. The effects of AG on SNI mice and its underlying mechanisms were examined by behavioral tests, qPCR, western blotting, ELISA, immunofluorescence (IF), ROS test, transmission electron microscopy and mitochondrial test.

**Results:**

We found that intraperitoneal administration of AG produced pronounced dose-dependent antinociceptive effects in SNI mice. Moreover, AG treatment significantly inhibited ERK, JNK and p38 phosphorylation in the lumbar spinal cord of SNI mice, but not AMPK, PGC-α and mTOR pathway. Meanwhile, we found that pretreatment with the U0126 or SB203580 or SP600125, 30 min prior to AG administration, blocked the analgesic effects of AG in SNI mice. Furthermore, mechanistic studies indicated that at the spinal cord level, AG produced pain relief through restoring mitochondrial biogenesis, inhibiting oxidative damage, suppressing microglia and astrocyte activation and decreasing the production of pro-inflammatory factors, which direct contributed to neuronal modulation. pretreating with minocycline reduced but did not completely block the analgesic effect of AG, indicating that the activation of spinal cord microglia is not necessary for the antiallodynic effect of AG. In addition to neuropathic pain, AG exhibits significant analgesic effects across diverse models, indicating its broad-spectrum analgesic properties. Concurrently, studies on short-term toxic side effects revealed that prolonged AG injection had no impact on hepatic or renal functions and produced none of the typical analgesic side effects, including tolerance, addiction, or constipation, indicating limited antinociceptive side effect.

**Conclusions:**

The present study is the first to provide evidences that AG may represent a novel therapeutic target with high analgesic activity and low side effects for the treatment of neuropathic pain.

## Graphical Abstract

**Figure:**
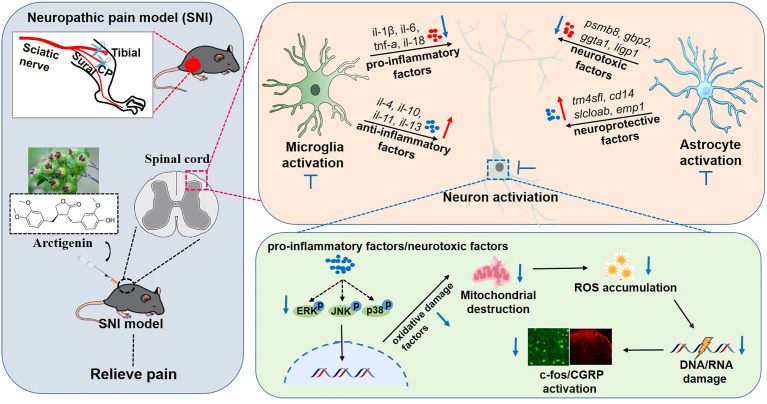
Potential mechanism of AG to ameliorate SNI-induced neuropathic pain in mice through inhibiting microglia activation and neuronal oxidative damage.

## 1 Introduction

Neuropathic pain is a common and challenging-to-treat condition triggered by disorders or injuries of peripheral nerves or the central nervous system (CNS) (1–3). Currently, according to International Association for the Study of Pain (IASP) guidelines, gabapentin and pregabalin are recommended as first-line treatments for neuropathic pain (1, 3, 4). However, pharmacovigilance databases have raised concerns about their potential for misuse and fatal overdose risks, particularly in patients with a history of substance abuse (5–7). Meanwhile, opioid analgesics serve as second-line therapy for neuropathic pain but are associated with long-term adverse effects such as tolerance development, addiction, respiratory depression, and gastrointestinal complications, which limit their widespread clinical application (8, 9). Therefore, identifying novel pain modulation targets holds significant importance for achieving comprehensive and enduring management of neuropathic pain.

Arctigenin (AG), a lignan compound extracted from plants such as *Arctium lappa (burdock)*, has garnered significant attention in recent years due to its notable antioxidant and anti-inflammatory activities (10–13). For instance, in terms of antioxidant activity, AG directly neutralizes reactive oxygen species (ROS) and reactive nitrogen species (RNS), including superoxide anion (O_2_^-^), hydroxyl radicals (·OH), and peroxynitrite (ONOO^-^), thereby reducing oxidative stress-induced damage to DNA, lipids, and proteins (14, 15). Additionally, AG enhances the transcriptional activity of the antioxidant response element (ARE) by promoting the nuclear translocation of nuclear factor erythroid 2-related factor 2 (Nrf2), which upregulates the expression of antioxidant enzymes such as superoxide dismutase (SOD), glutathione peroxidase (GSH-Px), and heme oxygenase-1 (HO-1) (16, 17). AG also protects cell membrane integrity by reducing the production of malondialdehyde (MDA), a terminal product of lipid peroxidation (15, 17). In anti-inflammatory studies, AG significantly reduced levels of TNF-α, IL-6, and nitric oxide (NO), while suppressing the expression of cyclooxygenase-2 (COX-2) and inducible nitric oxide synthase (iNOS) in lipopolysaccharide (LPS)-induced RAW264.7 macrophages (18). In collagen-induced arthritis mouse models, AG alleviated joint swelling and inflammatory infiltration by inhibiting the NF-κB and MAPK signaling pathways (19). Furthermore, AG downregulates inflammation-related gene expression by inhibiting the phosphorylation of p38, JNK, and ERK kinases (20). By suppressing COX-2 and iNOS, AG reduces the overproduction of prostaglandin E2 (PGE2) and NO, key mediators of inflammatory responses (19).

Neuropathic pain, neuroinflammation, and oxidative damage form a complex, interconnected network driven by multi-layered molecular mechanisms, creating a self-sustaining “pain-inflammation-oxidative stress” vicious cycle (21, 22). In neuropathic pain, activated microglia (central) and Schwann cells (peripheral) release pro-inflammatory cytokines such as TNF-α, IL-1β, and IL-6, which directly sensitize neurons (23, 24). Simultaneously, neurons in the spinal dorsal horn release chemokines like CCL2, recruiting monocytes to inflammatory sites and amplifying the inflammatory response (24). Additionally, studies report that in neuropathic pain, mitochondrial dysfunction and activation of NADPH oxidase (NOX) lead to excessive reactive oxygen species (ROS) production (25, 26). These ROS activate NF-κB and the NLRP3 inflammasome, promoting the maturation of IL-1β, disrupting neuronal membrane integrity, and releasing pain mediators such as prostaglandin E2 (PGE2) (27). For example, in chemotherapy-induced neuropathy, paclitaxel triggers ROS-mediated activation of TLR4 in microglia, leading to IL-1β release (28). In diabetic neuropathy models, the antioxidant N-acetylcysteine (NAC) reduces both spinal ROS levels and IL-6 expression (29).

Whether AG can exert an analgesic effect in neuropathic pain is unknown. Given AG’s demonstrated dual antioxidant and anti-inflammatory properties (13, 15, 17), it is critically important to investigate its potential role in modulating neuropathic pain and sought to uncover the underlying regulatory mechanisms. In this study, we found that intraperitoneal (i.p.) administration of AG produced pronounced and dose-dependent antinociceptive effects in SNI mice, indicating that AG may be involved in the regulation of neuropathic pain.

## 2 Materials and methods

### 2.1 Experimental mice

Adult male C57BL/6 mice (8-10 weeks, weight: 20-22 g) were purchased from the Animal Facility of Xuzhou Medical University for behavioral and biochemical experiments. All mice procedure in this study were approved by the Office of Laboratory Animal Research and the Institute of Animal Care and Use Committee of Xuzhou Medical University (approval number, 202208S014). All animals were group housed (22 ± 2 °C, 12 h light-dark cycle, 5 animals/cage) with water and food available *ad libitum*. All animals were used only once and all data were performed and analyzed by two independent experimenters blinded to the group assignment. Upon termination of the experiment, all animals were subjected to euthanasia using CO_2_. In detail, euthanize mice in the home cage to minimize the stress of being placed into an unfamiliar enclosure and to prevent social aggression. Place the mice’s home cage in the chamber where they are readily visible and can make normal postural adjustments. Introduce 100% CO_2_ into the chamber at a displacement rate of 30-70% of the chamber volume per minute. Use a pressure-reducing regulator and flow meter. Keep the mice in the CO_2_ for the 6 min. After exposure, verify each animal is dead using the method from your ACUC protocol (e.g., no gasping). If signs of life are present, return the animal to the CO_2_ chamber or use an approved secondary method.

### 2.2 Drugs

AG (CAS number: 7770-78-7) was purchased from MedChemExpress and dissolved in 10% DMSO. U0126 (inhibitor of ERK, cat number: HY-12031A), SB20358 (inhibitor of p38, cat number: HY-112349) and SP600125 (inhibitor of JNK, cat number: HY-12041) were purchased from MedChemExpress. Morphine sulfate was purchased from Shenyang First Pharmaceutical Factory (Shenyang, China). CFA (Sigma, F5881) and formalin (Sigma, 252547) were purchased from Sigma Chemical Company. Minocycline hydrochloride was purchased from Sigma and dissolved in saline. All drugs used in the behavioral tests were dissolved in sterilized saline and stored in 1.5 mL tubes at -20 °C.

### 2.3 Surgery of spared nerve injury model

The surgical procedure was performed as described by Decosterd and Woolf (30). Briefly, mice (22-25 g) were anesthetized with sodium pentobarbital (40 mg/kg, i.p., Sigma) and placed on the mini-operating table, and the sciatic nerve was exposed. Then the tibial and common peroneal branches of the sciatic nerve were ligated with 6.0 silk threads and 2-mm nerve portions were severed at distal side of the ligations, but the sural nerve was left intact. The mice were housed individually and allowed to recover for 7 days. All behavior experiments after AG injection (5, 10, 25 and 50 mg/kg, i.p.) were performed on 7 days after SNI.

### 2.4 Complete Freund adjuvant-induced peripheral inflammation pain model

According to previous reports (32, 33), CFA was used to induce peripheral inflammatory pain. 20 μL CFA was injected into the plantar surface of the right hind paw to induce inflammatory pain. The paw withdrawal thresholds were measured before inflammation and then at 1h, 2h, 4h and 24h. All behavior experiments after AG injection (5, 10, 25 and 50 mg/kg, i.p.) were performed on 3 days after CFA injections.

### 2.5 Assessment of pain behaviors

SNI and CFA model mice exhibited mechanical allodynia and thermal hyperalgesia. Therefore, mechanical pain and thermal pain are assessed using von Frey filaments and radiant heat tests, respectively.

#### 2.5.1 Mechanical allodynia

The von Frey filaments (Ugo Basile, Varese, Italy) were used to measure the mechanical thresholds of hind paws as described previously (35). In brief, the mice were placed on a metal mesh floor and covered by transparent plastic box. After at least 30 min habituation, drugs were administered, and the paw withdrawal threshold was measured by grade-strength von Frey monoflaments (2.36, 2.44, 2.83, 3.22, 3.61, 3.84, 4.08, 4.17 and 4.31) at 20, 40, 60, 90, and 120 min, respectively. In detail, the first von Frey filament (2.44) was applied to the plantar surface of the hind paw. If a withdrawal response was observed within 2 s, the filament (2.36) was used. Conversely, if the filament failed to evoke a withdrawal response, the next filament (2.83) was applied. A total of six responses were recorded, and the 50% paw withdrawal threshold (PWT) was statistically analyzed based on the up-down method (35, 36).

#### 2.5.2 Thermal hyperalgesia

Thermal pain was assessed using radiant heat tests. In summary, the mice were placed in a plexiglass chamber on a glass plate and allowed at least 30-60 min to habituate for three consecutive days. The Paw withdrawal latency (PWL)was recorded by an automatic timer and was defined as the duration from the onset of radiant heat focused to the withdrawal of the hind paw. In detail, a radiant heat source (IITC Life Science Model 390, 40W bulb) was applied to the mid-plantar surface of the hind paw. The heat beam intensity was adjusted at the beginning of the study to yield a baseline withdrawal latency of 15 ± 2 seconds in sham-operated mice. A cut-off time of 20 seconds was enforced to prevent potential tissue injury. For each mouse, with a 10-min interval, the heat stimulus was repeated three times to determine latency (34).

### 2.6 Formalin-induced acute inflammatory pain

The procedure in formalin-induced acute inflammatory pain had been reported previously (31). Briefly, mice were placed in a plexiglas chamber (50 × 50 × 40 cm^3^) and left to freely explore for a 30 min period. 5% formalin solution (20 μL) was i.pl. injected in the plantar area of the hind paw. AG (5, 10, 25 and 50 mg/kg) were i.p. injected 30 min prior to formalin injection. Subsequently, mice were immediately placed in a plexiglas chamber and recorded for 30 min. Then, the biphasic pain responses time of formalin test spent licking, shaking and biting the injected paw was recorded. First phase (phase I) is 0 - 5 min after formalin injection and second phase (phase II) is 15 - 30 min after formalin injection.

### 2.7 Capsaicin-induced nocifensive behaviors and secondary mechanical hyperalgesia

0.1% Capsaicin (W/V) was injected in the plantar area of the hind paw. Capsaicin was dissolved in 10% dimethyl sulfoxide (DMSO). Spontaneous pain behaviors (licking and paw flinching) were recorded over 15 min. In addition, capsaicin was injected subcutaneously into the lower hind leg to induce secondary mechanical hyperalgesia in the plantar area of the hind paw. The paw mechanical threshold was measured between 20-30 min and 45-60 min after capsaicin injection.

### 2.8 Acetic acid-induced visceral pain model

The procedure for the acetic acid-induced writhing test was described below. In brief, mice were placed in a plexiglas chamber (50 × 50 × 40 cm^3^) and left to freely explore for a 30 min period. 0.6% acetic acid (V/V, 10 mL/kg of body weight) were i.p. injected as an irritant stimulus. Then, AG (5, 10, 25 and 50 mg/kg) were i.p. administrated 5 min prior to acetic acid. Subsequently, the number of writhes was recorded for 5-15 min after acetic acid application. A writhe was defined as a contraction of abdominal musculature with an extension of hind limbs.

### 2.9 Antinociceptive tolerance test

The antinociceptive tolerance test was evaluated as described in our previous report (34). Briefly, SNI mice were allowed to recover for 7 days after surgery. In the antinociceptive tolerance study, the SNI mice were continuously injected saline, AG (25 mg/kg, i.p.), or morphine (10 mg/kg, i.p.) once daily for 8 days. Behavioral measurements were obtained 0-120 min after the drug injection on days 1, 4, and 8 using von Frey monofilaments.

### 2.10 Gastrointestinal transit test

The gastrointestinal transit (GIT) was carried out as described in our previous report (34). Briefly, mice were fasted for 18 h, i.p. injection of AG (25 and 50 mg/kg) after 10 min, and gavaged with 200 μL of charcoal marker (10% charcoal suspension in 5% gum arabic solution). The mice were killed at 30 min after gavage, and the small intestine was removed. The distance traveled by the charcoal marker was measured.

### 2.11 Locomotor activity test

The open field was adopted as the model of exploring the locomotor activity of mice and conducted as previously reported (34). The open field was a square arena (50 × 50 × 40 cm^3^) with a black floor and Plexiglas walls, and the open field was illuminated by overhead fluorescent lighting (120 lx). Mice-injected saline or AG (25 and 50 mg/kg, i.p.) were placed in the center of the open field and left to freely explore for a 30 min period, and the operator needs to sit approx. 1.5 m away from the apparatus in this period. Locomotor activity was measured by the open field test analysis system (TME, Chengdu, China). The box was cleaned between each experiment with 96% absolute ethanol, and all experiments were conducted between 10:00 a.m. and 6 p.m.

### 2.12 Conditioned place preference

The conditioned place preference (CPP) experiment typically consists of three phases: pretest, conditioning and test phase. The apparatus consists of two spacious chambers (20 × 20 × 20 cm^3^) connected by a narrow passage (5 × 20 × 20 cm^3^). The two main chambers (white-colored walls with a coarse floor compared to black-colored walls with a smooth floor) present distinct visual and tactile cues and can be isolated from each other by a guillotine door. In pretest (day 1), mice are placed in the apparatus for 15 minutes, and the time spent in each chamber is recorded. Mice exhibiting an inherent preference for either chamber exceeding 60% are excluded from the study. During the conditioning phase (days 2-4), which lasts for three consecutive days, mice receive a daily i.p. injection of saline and are confined to one designated chamber for 40 minutes by closing the guillotine door. After a 6-hour interval, the mice receive daily injections of saline, morphine, or AG (25 and 50 mg/kg) intraperitoneally and are confined to the opposite chamber for 40 minutes. This process associates specific chambers with the administered substances. In the test phase (day 5), mice are placed in the apparatus and allowed to move freely throughout all chambers for 15 minutes. The time spent in each chamber is recorded to assess the drug’s effect on preference for the context-associated chamber. The conditioned place preference score was quantified as the time stayed in the drug-related chamber on day 5 minus the time stayed in the drug-related chamber on day 1.

### 2.13 Immunofluorescence

The SNI mice received single i.p. injection of saline or AG. 30 min after the injection, the mice were deeply anesthetized with sodium pentobarbital (40 mg/kg, i.p.), and then transcardially perfused with 20 ml PBS followed by 40 ml 4% paraformaldehyde. After the perfusion, the L4 to L5 spinal cord was removed and postfixed in 4% paraformaldehyde overnight. For the immunofluorescence experiment, the samples were dehydrated with 30% sucrose solution, imbedded, and sliced into consecutive frozen sections (15 μm). Sections were blocked with 5% donkey serum containing 0.1% Triton for 2 h and incubated at 4 °C overnight with primary antibodies (**Supplementary Table S1**). After washing twice with PBS for 10 min, the sections were incubated with the secondary antibody (Cy3-labeled goat anti-mouse immunoglobulin G [IgG] and fluorescein isothiocyanate [FITC]-labeled goat anti-rabbit IgG) for 2 h at room temperature. Images were captured using a 20x objective lens on a confocal microscope (FluoView1000; Olympus, Japan), resulting in a final magnification of 200x. All image analyses were performed using ImageJ (Fiji) software (National Institutes of Health). Signal Intensity (for markers like 8-OHdG): regions of interest (ROIs) were drawn. After uniform background subtraction, the mean fluorescence intensity (MFI) was measured. Data are expressed as relative fluorescence intensity. Cell Counting (for c-Fos+, Iba1+, GFAP+ cells): Positive cells were identified based on co-localization of a clear nucleus (DAPI) and specific fluorescence signal above a set threshold. They were manually counted. The unit of measurement for cell counts (for c-Fos+, Iba1+, and GFAP+ cells) are presented as the number of positive cells per field. Fiji Image J software was used to provide a quantitative measurement of the co-localization of 8-OHdG with NeuN or iba1 by Pearson’s correlation coefficient, which estimated the degree of overlap between fluorescence signals obtained in two channels. The degree of co-localization from the Pearson’s coefficient values was categorized as very strong (0.85 to 1.0), strong (0.49 to 0.84), moderate (0.1 to 0.48), weak (-0.26 to 0.09) based on previously reports (37, 38).

### 2.14 Quantitative polymerase chain reaction

Under deep anesthesia, the L4-L5 spinal cord segments of mice were quickly removed and analyzed. Total RNA was isolated with TRIzol reagent ((Vazyme, R401-01) according to manufacturer’s instructions. cDNA was then synthesized using the 5X PrimeScript RT Master Mix (TaKaRa). Quantitative realtime polymerase chain reaction (TaKaRa) was performed in a 20 µL reaction mixture consisting of 10 µL of 2X SYBR Premis Ex TaqTM II, 2 µL of cDNA, 1 µL of forward primer, 1 µL of reverse primer and 6 µL of ddH2O. The thermal cycle conditions used were 95˚C for 5 min, 40 cycles of 95˚C for 5 s and 58˚C for 30 s, and 72 °C for 30 s. Specific primers used for the detection were as followed in **Supplementary Table S2**. Relative mRNA levels were calculated using the 2-ΔΔCT method (39). Gene expression was first normalized to the housekeeping control gene GAPDH.

### 2.15 Western blotting

The ipsilateral side of L4-L5 spinal cord segments were quickly removed from deeply anesthetized mice and stored at -80 °C. The tissues were homogenized in RIPA lysis buffer containing a cocktail of protease inhibitor (Servicebio, Wuhan). The protein concentrations of the lysates were estimated using the method of BCA (Thermo Scientific, 23225) and the total protein content between samples was equalized. The total protein was separated by SDS-PAGE and electrophoretically transferred onto polyvinylidene fluoride membranes (PVDF, Millipore, IPV H00010). PVDF membranes were blocked with 5% fat-free milk (w/v) for 2 h and incubated with specific primary antibodies at 4 °C overnight, the following primary antibodies were used in **Supplementary Table S1**. The membranes were then incubated peroxidase-conjugated secondary antibodies (Servicebio, Wuhan). The membrane was detected with ECL substrate (Thermo Scientific, 32106), and exposed using a ChemiDoc XRS imaging system (Bio-Rad, USA). The intensity of blots was quantified by the Image J software (National Institutes of Health, Bethesda, MD, USA).

### 2.16 Enzyme-linked immunoassay

In the acute toxicity experiment, mice intraperitoneally administered high-dose AG (50 mg/kg) for one consecutive week were subjected to blood collection for ELISA analyses. At 60 minutes following the last AG administration, total proteins in plasma were taken from experimental mice. Liver function factors (AST, ALT, ALP, ALB, TBA, TBIL), kidney function factors (UA, CREA, UREA), lipid profile factors (CHOL, HDL-C, LDL-C, TG), cardiac enzyme profile factors (CK, CK-MB, HBDH, LDH) and glucose in plasma were quantified using commercially available ELISA kits (Servicebio, Wuhan), according to the manufacturer’s instructions.

### 2.17 Hematoxylin-eosin staining

In the acute toxicity experiment, mice intraperitoneally administered high-dose AG (50 mg/kg) for one consecutive week were subjected to tissue sampling (liver, kidneys, spleen, lungs) for H&E staining analyses. At 60 minutes following the last AG administration, mice were deeply anesthetized and intracardially perfused with 20 mL PBS followed by 20 mL 4% paraformaldehyde (PFA). Liver, spleen, kidney and lung were removed and postfixed in 4% paraformaldehyde overnight. The H&E staining procedures were described in detail in our previous report (40), coronal paraffin sections (5 μm) were prepared and stained with H&E staining kit (cat number: C0105, Beyotime, Shanghai, China) using standard methods. The images were processed by ordinary optical microscope (H&E) (ZEISS International, optical and optoelectronic technology, USA).

### 2.18 Transmission electron microscopy

One weeks after SNI, mice received heart perfusion with 4% paraformaldehyde after deep anesthesia and saline perfusion, lumbar cord tissue (L4-L5) was isolated and excised, then cut to 1 × 3 mm and fixed in 2.5% buffered glutaraldehyde and 1% osmium tetroxide (Servicebio) for 48 h, and then dehydrated by alcohol gradient and acetone before the tissue was embedded in resin. The tissue was sliced into 0.05 μm-thick sections using a Shanghai Leica instrument Co., Ltd. (Pathology slicer, Shanghai China). Finally, sections from each group were imaged using a transmission electron microscope (Hitachi HT7700Hitachi, Tokyo, Japan) after contrast-staining with uranyl acetate and lead citrate.

### 2.19 Statistical analysis

In this experiment, statistical software SPSS 19.0 (IBM Corp., Armonk, NY, USA) was used for statistical analysis of data. All data are presented as mean ± standard error (mean ± SEM). Comparison of data between different groups was conducted using one-way ANOVA followed by bonferroni *post hoc* tests. Comparisons of time-series data were done with two-way repeated measures ANOVA. Statistical analysis of the behavioral data used in this experiment were as followed in **Supplementary Table S3**. The *p*-values < 0.05 was considered to be statistically significant.

## 3 Results

### 3.1 AG treatment produced an antinociceptive effect in SNI mice

To investigate the potential role of AG in neuropathic pain, mechanical and thermal paw withdrawal thresholds were measured on days 1, 3, 7 and 14 after SNI induction. Behavioral tests showed that mechanical allodynia and thermal hyperalgesia in SNI mice began on day 3, and the mice showed stable mechanical and thermal pain thresholds on day 7 (data not shown).

**Figure 1:**
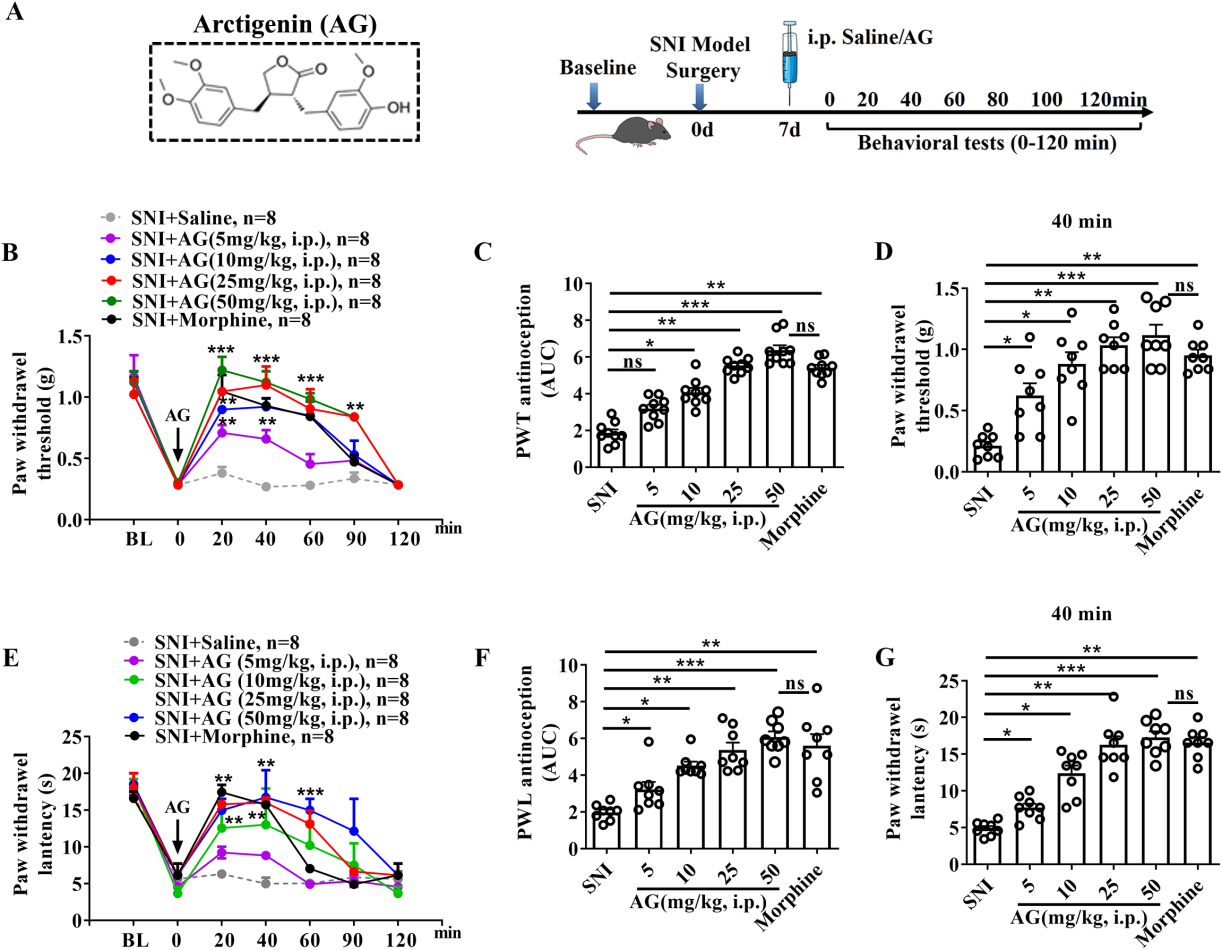
AG treatment attenuates SNI-induced mechanical pain hypersensitivity and thermal sensitivity. **(A)** Experimental design. **(B, E)** Antinociceptive dose- and time-response curve for i.p. administration of AG (5, 10, 25 and 50 mg/kg) in SNI mice. **(C, F)** The extent and duration of analgesia are estimated by the area under curve (AUC (g min)) of PWT vs time (0-120 minutes). **(D, G)** Mechanical allodynia and thermal allodynia were tested 40 min after administration of AG (i.p.). Data are expressed as the Mean ± SEM, n = 8-9 for each group. *p < 0.05, **p < 0.01, and ***p < 0.001 compared with SNI group. The "ns" indicates no significant difference.

**Figures 1A** shows AG's chemical structure and experimental timeline. In von Frey monofilaments tests, before AG administration, the mice exhibited stable mechanical pain thresholds (withdrawal threshold: 0.224 ± 0.32 g). In contrast, AG treatment (5, 10, 25 and 50 mg/kg, i.p.) significantly elevated mechanical withdrawal thresholds in SNI model mice, with effects peaking approximately 20 min after AG application (5 mg/kg: 0.54 ± 0.09 g, 10 mg/kg: 9.13 ± 0.06 g, 25 mg/kg: 0.99 ± 0.06 g, 50 mg/kg: 1.19 ± 0.14 g, all *p* < 0.05) (**Figure 1B**). As shown in **Figure 1C**, the extent and duration of analgesia were estimated according to the AUC values. The AUCs were calculated during 0-120 min from the AG dose-response curve using trapezoidal rules and were analyzed using one-way ANOVA followed by bonferroni’s *post-hoc* test. The data suggested that AG treatment induced dose-dependent antinociceptive effects in SNI mice (*p* < 0.05 for 10 mg/kg AG group vs SNI group, *p* < 0.01 for 25 mg/kg AG group vs SNI group, *p* < 0.001 for 50 mg/kg AG group vs SNI group, **Figure 1C**). Meanwhile, the i.p. injection of morphine (10 mg/kg) also showed analgesic effects against mechanical hyperalgesia in SNI mice (*p* < 0.01 for morphine group vs SNI group, **Figure 1C**). The extent and duration of the analgesic effects of AG (25 mg/kg and 50 mg/kg) were greater than those of morphine; however, the difference was not significant (**Figures 1B–D**). The dosage of morphine used referred to previous studies and our observations (31).

Similarly, in thermal hyperalgesia tests, i.p. administration of AG produced a dose-dependent analgesic effects in SNI mice, with peak efficacy observed at 20 min post-injection (15.3 ± 0.6 s vs baseline 4.5 ± 0.4 s, **Figure 1E**), and complete cessation of activity by 120 min. Both 25 mg/kg and 50 mg/kg doses demonstrated analgesic efficacy comparable to morphine (**Figures 1F, G**). Additionally, we examined the effects of AG in female SNI mice. As shown in **Supplementary Figure S1**, i.p. administration of AG also produced dose-dependent analgesic effects in the female SNI mouse model (*p* < 0.01 for 10 mg/kg and 25 mg/kg of AG, *p* < 0.001 for 50 mg/kg of AG and morphine, **Supplementary Figure S1A**). A comparison of mechanical pain thresholds between male and female mice 30 minutes after AG injection revealed no sex-dependent differences in its analgesic effects (**Supplementary Figure S1C**). It should be noted that the mechanistic investigations were conducted only in male mice, limiting insight into potential sex-specific mechanisms. This represents an important direction for future research.

### 3.2 Identification of transcriptome sequencing regulated by AG in spinal cord tissue

**Figure 2:**
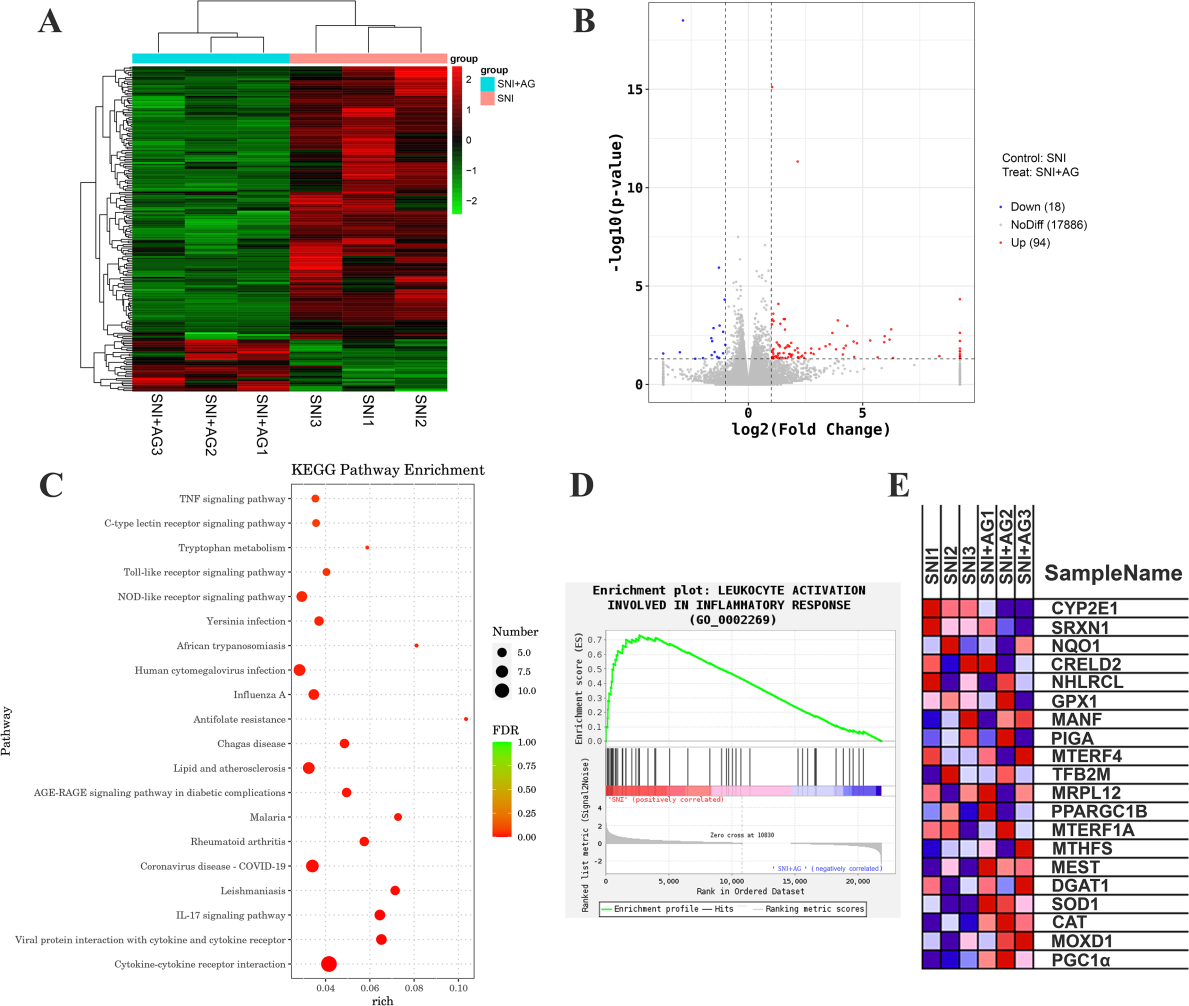
Transcriptome sequencing of spinal cord tissue in SNI mice after AG treatment. **(A)** Heat map depicting differentially expressed genes in SNI with or without AG **(B)** Volcano plots depicting the differential abundance of AG treatment. **(C)** KEGG pathway analysis. **(D, E)** Genes related to inflammatory response and antioxidant metabolism significantly up-regulated and down-regulated in RNAseq analysis (*p* < 0.01). The Log2 fold-change represents the ratio of the base mean (the average expression levels of samples were compared after standardization) between SNI and SNI+AG. The redder colour of the Log2 fold-change indicates a higher level of gene expression (n = 3).

Next, to profile the transcriptome regulated by AG, we subjected SNI with or without AG to RNA sequencing (RNA-seq). AG treatment resulted in 1247 genes altered globally, including 94 up-regulated genes and 18 down-regulated genes (**Figures 2A, B**). Volcano plots depicting the differential abundance of AG treatment (**Figure 2B**). Kyoto Encyclopedia of Genes and Genomes (KEGG) pathway analysis of the differentially expressed genes (DEGs) after AG treatment showed enriched biological functions, including ‘TNF signaling pathway’, ‘cytokine-cytokine receptor interaction’, ‘inflammatory response’ and ‘lipid and atherosclerosis’ (**Figure 2C**), consistent with its role in antioxidant and anti-inflammatory activities (**Figure 2D**). RNAseq gene expression analysis shown gene sets involved in anti-oxidant and anti-inflammatory (CYP2E1, NQO1, MANF, SRXN1, PPARGC1B, GPX1, SOD1, CAT, and PGC1α, **Figure 2E**) were screened in response to AG management. Taken together, these results supported the analgesic effect of AG in neuropathic pain may be related to the regulation of oxidative stress or inflammatory responses.

### 3.3 AG suppressed microglia and astrocyte activation and pro-inflammatory cytokine production in the spinal cord

**Figure 3:**
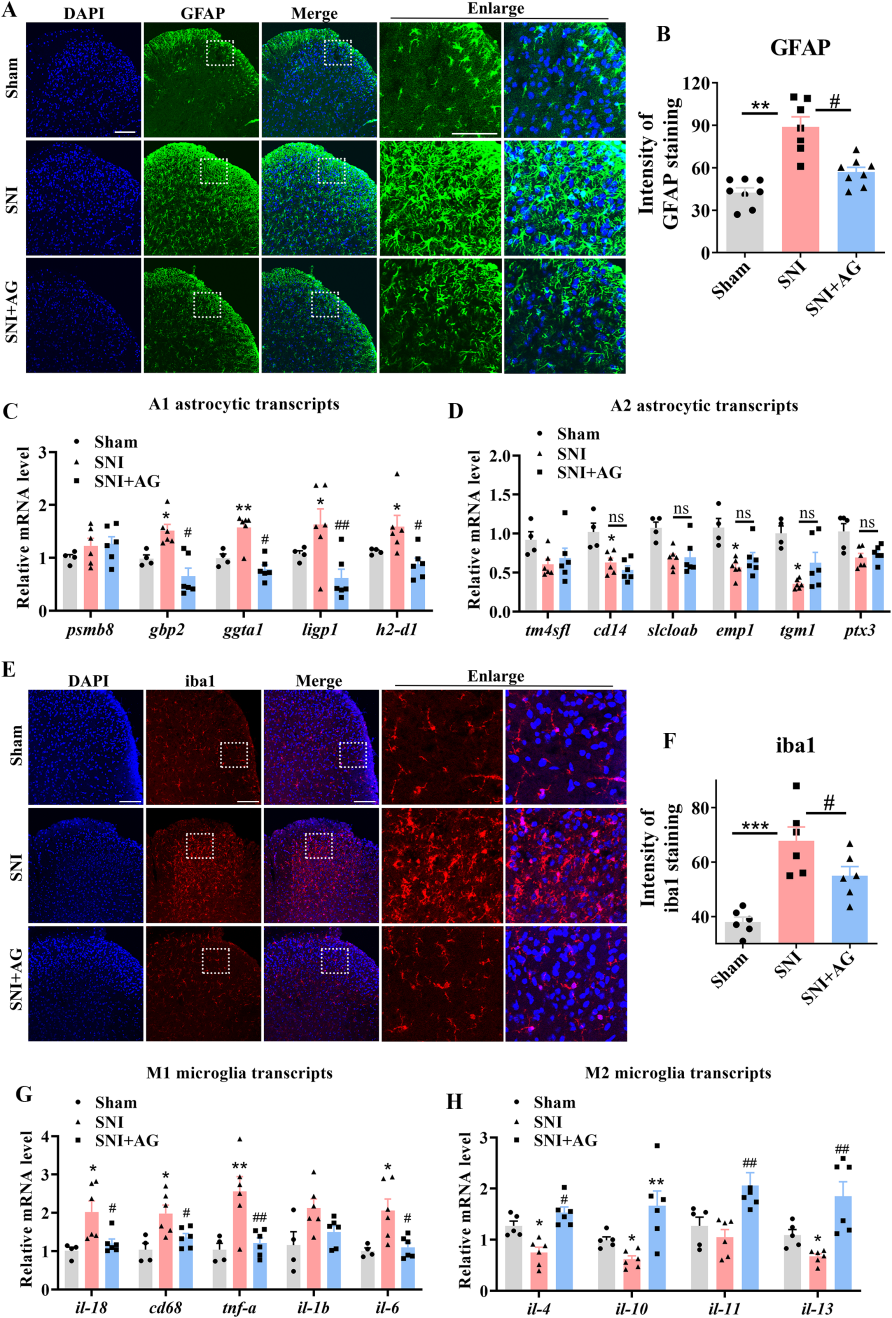
AG suppresses SNI-induced microglial and astrocyte activation and the production of pro-inflammatory cytokine in lumbar spinal dorsal horn. **(A)** Representative images show the effect of AG on activation of astrocytes (GFAP, green) 7 days after surgery. n = 6-8, scale bar = 100 μm. **(B)** Data summary of GFAP positive cells (n = 6-8). **(C)** The expression of A1 astrocyte marker (*psmb8, gbp2, ggta1, ligp1* and *h2-d1*) and **(D)** A2 astrocyte marker (*tm4sfl, cd14, slcloab, emp1, tgm1* and *ptx3*) were measured in the spinal cord in sham, SNI, and SNI + AG mice by qPCR (n = 6). **(E)** Representative images show the effect of AG on activation of microglia (iba1, red) 7 days after surgery. n = 6, scale bar = 100 μm. **(F)** Data summary of iba1 positive cells (n = 6). **(G)** The expression of M1 microglial marker (*il-1β, il-6, tnf-a, cd68* and *il-18*) and **(H)** M2 microglial marker (*il-4, il-10, il-11* and *il-13*) were measured in the spinal cord in sham, SNI, and SNI + AG mice by qPCR (n = 6). Data are expressed as the Mean ± SEM. *p < 0.05, **p < 0.01, and ***p < 0.001 for sham group vs SNI group. #*p* < 0.05, and ##*p* < 0.01 for SNI group vs AG+SNI group. The "ns" indicates no significant difference.

Based on transcriptome sequencing (RNA-seq) data and given the crucial roles of glia in neuropathic pain (41–44), we further investigated whether microglia and astrocyte activation was necessary for the antiallodynic effect of AG. As shown in **Figure 3**, 7 days after SNI surgery, the number of iba1-positive microglia and GFAP-positive astrocytes in the spinal dorsal horn, were dramatically activated in the SNI group compared with that in the sham group (*p* < 0.01 for **Figures 3A, B**, p < 0.001 for **Figures 3E, F**). Then, AG administration (25 mg/kg, i.p.) significantly suppressed microglia and astrocyte activation in SNI mice (*p* < 0.05 for **Figures 3B, F**), when compared with saline administration. Several studies have shown that activated microglia and astrocyte are the main source of pro-inflammatory cytokines in the spinal cord (45–47).

We further sought to evaluate the phenotype of spinal cord microglia and astrocyte in mice administered AG after neuropathic pain. The qPCR results showed that AG administration attenuated SNI-induced increases in A1 astrocyte marker *psmb8, gbp2, ggta1, ligp1* and *h2-d1* expression levels (*p* < 0.05 for *gbp2, ggta1*, and *h2-d1*; *p* < 0.01 for *ligp1*; **Figure 3C**), whereas the level of A2 astrocyte marker *tm4sfl, cd14, slcloab, emp1, tgm1* and *ptx3* were not changed in AG treated mice at 7 days after SNI (*p* > 0.05 for *tm4sfl, cd14, slcloab, emp1, tgm1 and ptx3;***Figure 3D**). Meanwhile, we measured the expression levels of microglia marker in the spinal cord using qPCR. The results found that increasing AG treatment significantly downregulated levels of M1 microglial marker *il-6, tnf-a, cd68* and *il-18* after SNI (*p* < 0.05 for *il-6, cd68 and il-18*; *p* < 0.01 for *tnf-a*; **Figure 3G**), while the mRNA level of *il-4, il-10, il-11* and *il-13*, which are produced by M2 microglia were elevated in AG injected mice on day 7 after SNI compared with saline injected mice (*p* < 0.05 for *il-4*; *p* < 0.01 for *il-10, il-11* and *il-13*; **Figure 3H**). These findings suggested that, following SNI, AG exerts its antinociceptive effects by inhibiting pro-inflammatory cytokine production in the spinal cord dorsal horn.

### 3.4 AG inhibited oxidative damage in the spinal cord

**Figure 4:**
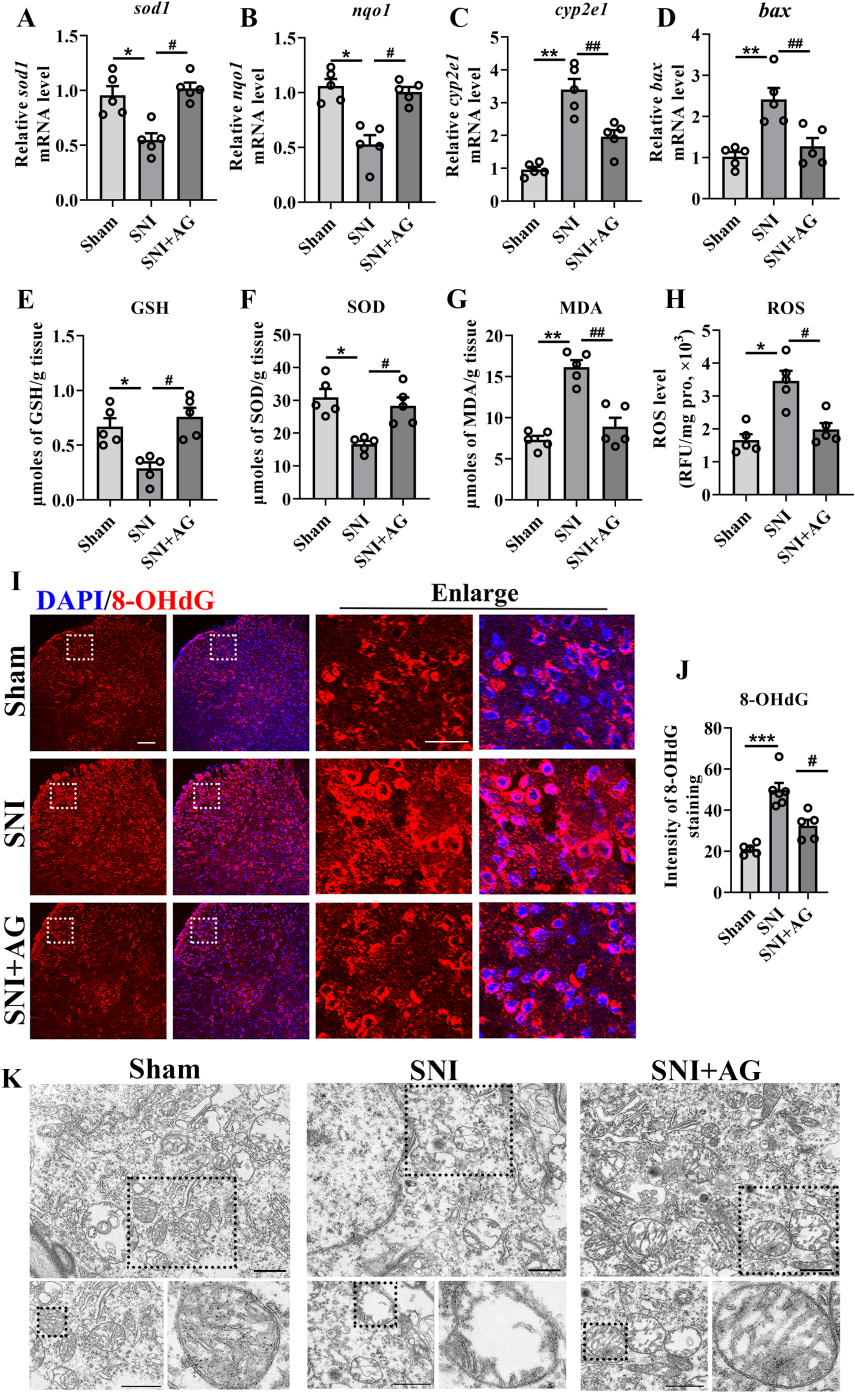
AG improves SNI-induced mitochondrial dysfunction. **(A–D)** The expression of *sod1, cyp2e1, nqo1*, and *bax* were measured in the spinal cord by qPCR (n = 5). **(E–H)** The levels of GSH, SOD, MAD, and ROS were measured by Elisa kits. **(I)** The expression of 8-OHdG in the spinal dorsal horn (Scale bar = 100 μm). **(J)** Qualitative data showed the intensity of 8-OHdG in the spinal dorsal horn (n = 5). **(K)** Representative picture of mitochondrion (dotted box) in neurons by transmission electron microscopy in the spinal dorsal horn. Scale bar = 1 μm. Data are expressed as the mean ± SEM. *p < 0.05, **p < 0.01 and ***p < 0.001 for sham group vs SNI group. #*p* < 0.05, and ##*p* < 0.01 for SNI group vs AG+SNI group.

Based on transcriptome sequencing (RNA-seq) data and given the crucial roles of mitochondrial oxidative damage in neuropathic pain, we further evaluated the effect of AG on oxidative damage in spinal cord. As shown in **Figures 4A–D**, qPCR results found that the expression of antioxidant factor such as superoxide dismutase (*sod1*) and NAD(P)H dehydrogenase quinone1 (*nqo1*) were significantly decreased in SNI mice (*p* < 0.05 for *sod1* and *nqo1*, **Figures 4A, B**). With exposed to AG, the expression of *sod1* and *nqo1* were remarkably elevated by about 50% (*p* < 0.05 for *sod1* and *nqo1*, **Figures 4A, B**). Then, the up-regulation of genes related to oxidative damage factors such as cytochrome P4502E1 (*cyp2e1*) and BCL2-associated X protein (*bax*) were suppressed remarkably by AG treatment (*p* < 0.01 for *cyp2e1* and *bax*, **Figures 4C, D**). Meanwhile, ELISA results observed that AG treatment significantly enhanced SNI-induced GSH and SOD production (*p* < 0.05 for GSH and SOD, **Figures 4E, F**), and inhibited SNI-induced MDA and ROS production (*p* < 0.01 for MDA, *p* < 0.05 for ROS, **Figures 4G, H**).

It’s been extensively documented that 8-hydroxydeoxyguanosine (8-OHdG), an oxidized nucleoside of DNA, was the most frequently reflected the ROS level and DNA lesion in nuclear and mitochondrial (48, 49). In this study, we found that the expression levels of 8-OHdG were significantly upregulated 7 days after SNI (*p* < 0.001, **Figures 4I, J**), whereas the opposite was seen with AG administration (*p* < 0.05, **Figures 4I, J**), suggesting that AG treatment inhibited SNI-induced ROS and oxidative damage. Subsequently, to assess the effects of AG in mitochondrial damage, transmission electron microscopy was performed to observe mitochondrial morphology. The results revealed that mitochondrial cristae were lost and the size and perimeter of mitochondria were decreased in SNI mice (**Figure 4K**). Whereas the AG treatment group was similar to that of the sham group, the mitochondria contained a complete inner membrane, an outer membrane, and cristae in an oval shape (**Figure 4K**).

### 3.5 AG-mediated oxidative damage localizes to neuron and attenuates neuronal activation in the spinal cord

**Figure 5:**
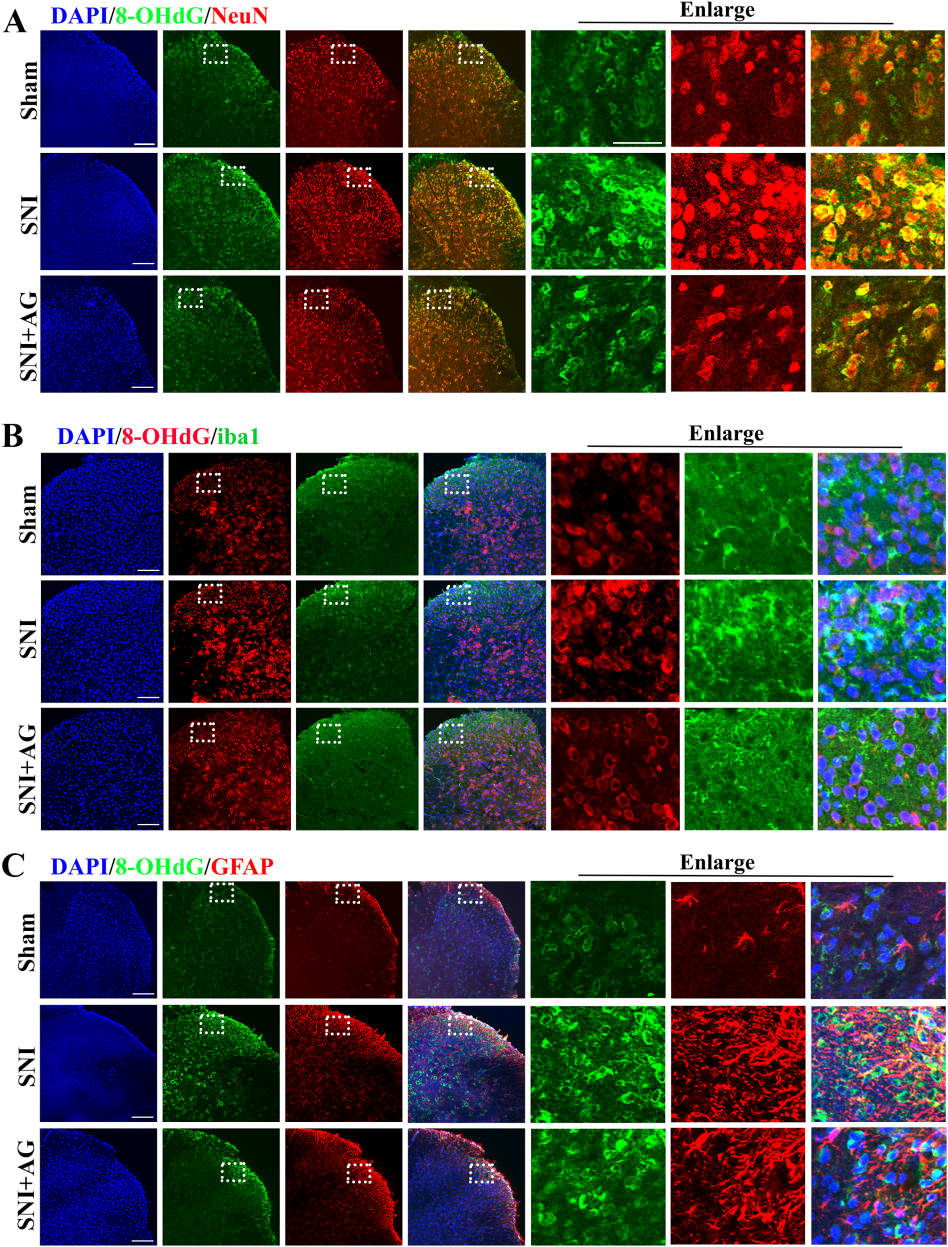
AG-mediated oxidative damage localizes to neuron. **(A)** Immunofluorescence showed the co-localization of 8-OHdG (green) with neurons (NeuN, red) in the spinal dorsal horn (Scale bar = 100 μm). **(B)** The co-localization of 8-OHdG (red) with microglial cells (iba1, green) in the spinal dorsal horn (Scale bar = 100 μm). **(C)** The co-localization of 8-OHdG (green) with astrocytes (GFAP, red) in the spinal dorsal horn (Scale bar = 100 μm).

To analysis of the cellular distribution revealed that ROS-induced DNA oxidative damage was distributed throughout microglia, astrocyte or neuron in the spinal dorsal horn. We performed double immunofluorescence staining for 8-OHdG and microglial marker (iba1), astrocyte marker (GFAP) or neuronal marker (NeuN) in the spinal dorsal horn. As shown in **Figures 5A–C**, confocal images showed that 8-OHdG co-localized primarily with NeuN (**Figure 5A**), although some overlap with iba1 and GFAP were also detected (**Figures 5B, C**). Image J software was used for the quantification of co-localization according to Pearson’s correlation coefficient. Collectively, these results suggested that AG inhibits oxidative damage mainly in neurons in the spinal dorsal horn of SNI mice.

**Figure 6:**
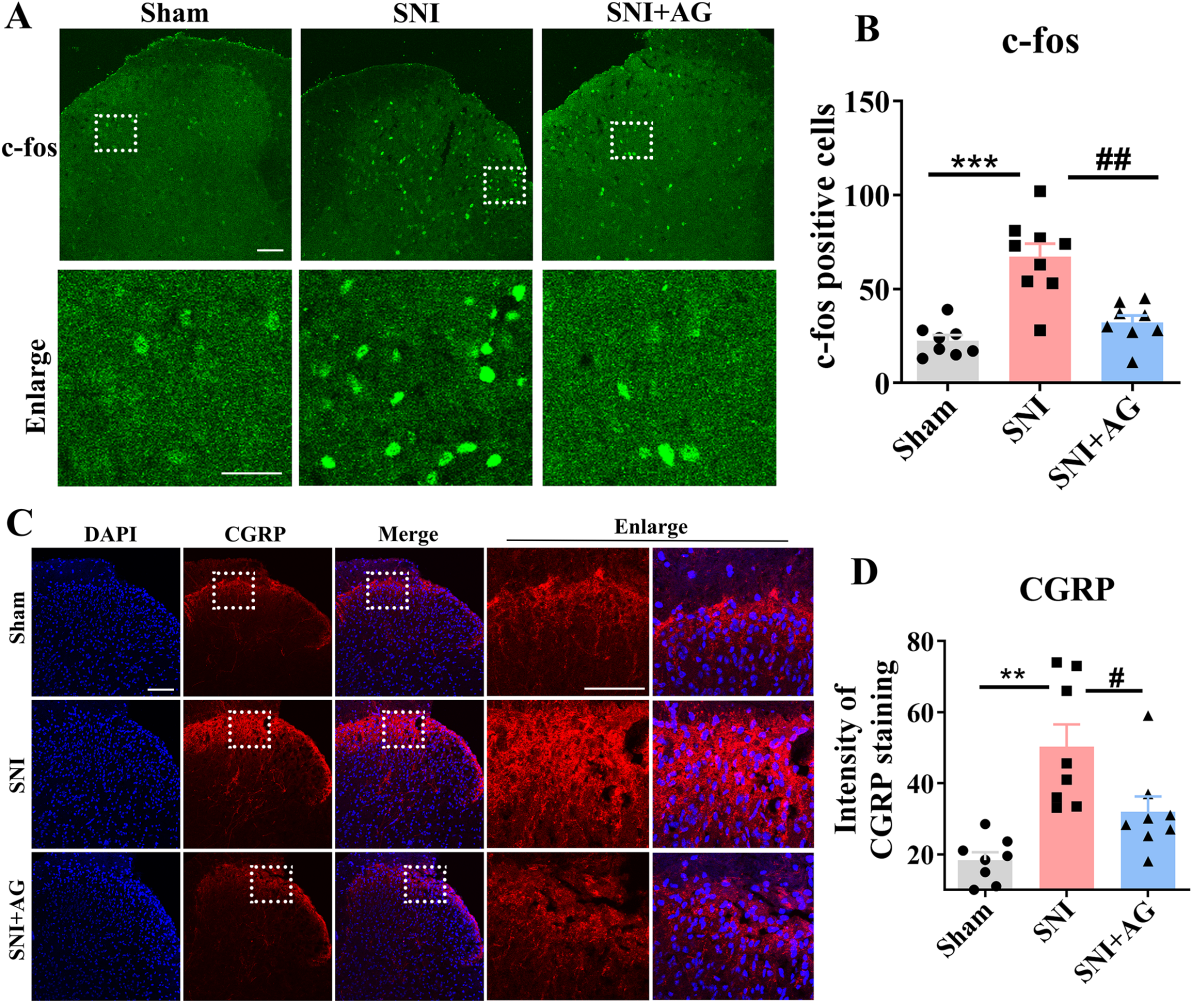
AG inhibites activation of c-fos and CGRP sensory neurons in SNI mice. **(A)** Representative images of c-fos immunofluorescence (green) in the spinal dorsal horn (scale bar 100 = μm). **(B)** Qualitative data showed the number of c-fos positive neurons in the spinal dorsal horn (n = 8). **(C)** Representative images of CGRP immunofluorescence (red) on the spinal dorsal horn (scale bar = 100 μm). **(D)** Qualitative data showing the intensity of CGRP positivity in the spinal dorsal horn (n = 6). Data are expressed as the mean ± SEM. **p < 0.01, and ***p < 0.001 for sham group vs SNI group. #*p* < 0.05, and ##*p* < 0.01 for SNI group vs AG+SNI group.

Subsequently, we examined the effects of AG on the activation of spinal cord neurons. The expression of c-fos in the dorsal horn of the spinal cord is a well-recognized marker for neuronal activity following peripheral stimulation. As shown in **Figures 6A, B**, 7 days after SNI induction, the number of c-fos positive neurons was significantly increased in the spinal cord dorsal horn of mice in the SNI group relative to that in animals in the sham group (*p* < 0.001, **Figures 6A, B**). In contrast, a significant reduction in the number of c-fos-positive neurons was observed following AG treatment (*p* < 0.01, **Figures 6A, B**). Furthermore, numerous studies have documented that the excitation of CGRP-expressing nociceptive sensory neurons are exhibited in both central and peripheral nervous systems under neuropathic pain conditions (50, 51). Thus, we examined the expression of CGRP in the spinal cord dorsal horn of SNI mice. As shown in **Figures 6C, D**, immunofluorescence experiment revealed that SNI treatment significantly enhanced the expression of CGRP in the spinal cord dorsal horn compared with the sham group (*p* < 0.01, **Figures 6C, D**). Whereas AG treatment resulted in significant down-regulation of CGRP expression (*p* < 0.05, **Figures 6C, D**).

**Figure 7:**
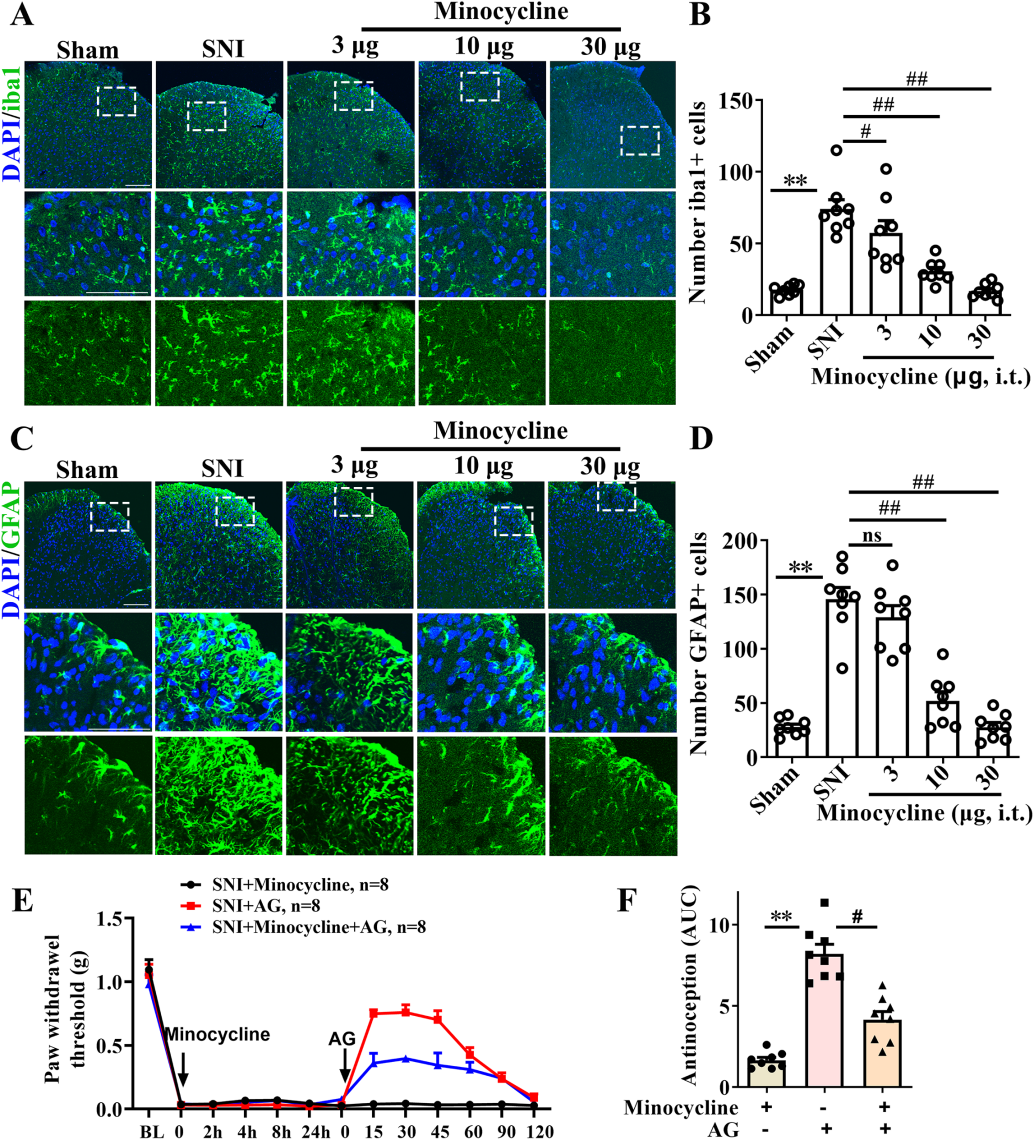
Inhibition of spinal dorsal horn glial cell activation is not necessary for the antiallodynic effect of AG. **(A)** Effect of minocycline treatment (i.t., 3, 10 and 30 μg) in SNI-induced the activation of microglia on the dorsal horn (scale bar 50 μm, n = 5-6). **(B)** Qualitative data showing the number of iba1+ cells in the dorsal horn. **(C)** Effect of minocycline treatment (i.t., 3, 10 and 30 μg) in SNI-induced the activation of astrocytes on the dorsal horn (scale bar 50 μm, n = 5-6). **(D)** Qualitative data showing the number of GFAP+ cells in the dorsal horn. **(E)** Pretreatment with minocycline (10 μg, i.t.) 24 h before the i.p. administration of AG reduced but did not completely block the analgesic effect of AG. n = 8 per mouse. Results are expressed as the Mean ± SEM. **p < 0.01 compared with SNI group. #*p* < 0.05 and ##*p* < 0.01 compared with AG+SNI group. The "ns" indicates no significant difference.

Next, we considered whether the effect of AG on spinal dorsal horn neurons requires the involvement of glial cells. To test this hypothesis, we used minocycline, an inhibitor of microglial and astrocyte activation, to investigate whether glial cells are an essential step in the mechanism of AG’s action. As shown in **Figure 7**, i.t. administration of minocycline (3, 10, and 30 μg) significantly suppressed the activation of both microglia and astrocytes (*p* < 0.05 for iba1 and GFAP, **Figures 7B, D**), with doses of 10 and 30 μg restoring activation levels to those observed in the normal control group (**Figures 7B, D**). Subsequently, by pretreating with minocycline (10 μg, i.t.) 24 hours before the i.p. administration of AG, we found that minocycline reduced but did not completely block the analgesic effect of AG (*p* < 0.05 for SNI + AG vs SNI + Minocycline + AG, **Figure 7F**). Additionally, at the 10 μg dose, minocycline itself had no effect on pain threshold. These data indicate that the inhibition of spinal dorsal horn glial cell activation is involved in the analgesic effect of AG, but it is not an essential, obligatory step.

### 3.6 Pharmacological inhibition of MAPK attenuates the analgesic effect of AG in SNI mice

**Figure 8:**
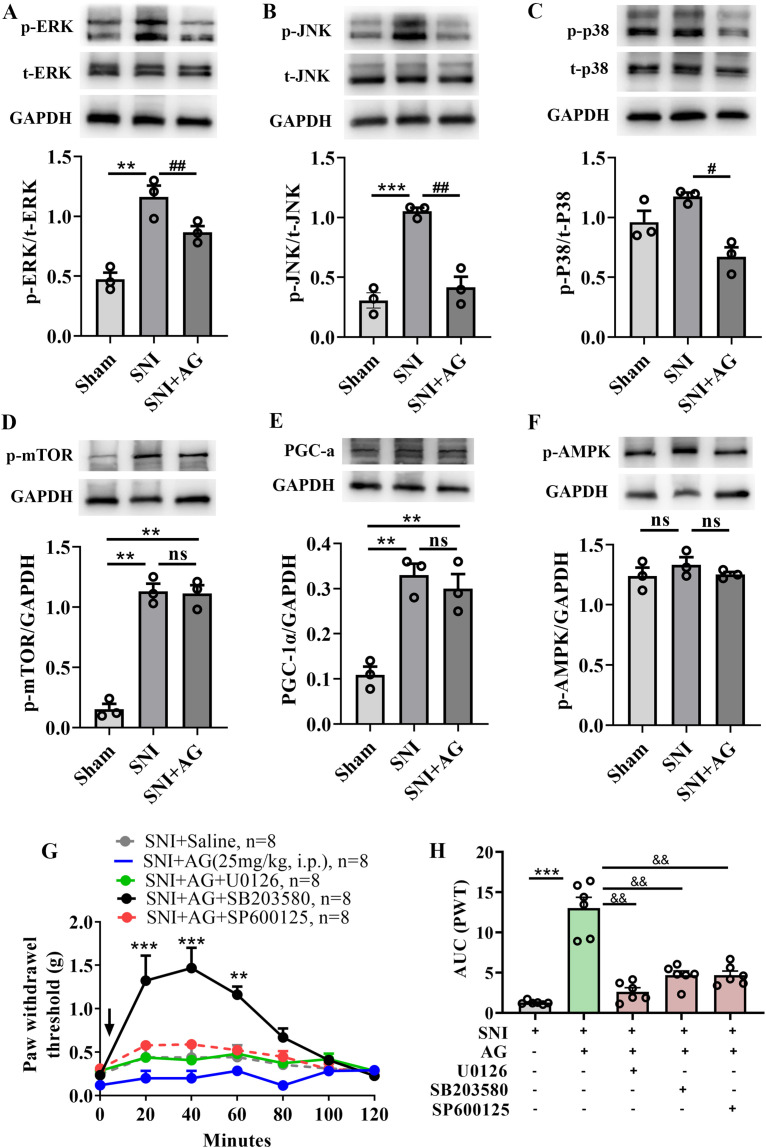
The antiallodynic effects of AG in SNI mice are associated with MAPK signaling pathway. **(A)** A shows western blotting of p-ERK and t-ERK in each group of spinal cord and qualitative data of protein expression in different groups. **(B)** Western blotting of p-JNK and t-JNK in different groups. **(C)** Western blotting of p-p38 and t-p38 in different groups. **(D)** Western blotting of p-mTOR in different groups. **(E)** Western blotting of PGC-a in different groups. **(F)** Western blotting of p-AMPK in different groups. **(G)** The analgesic effects of AG in SNI mice could be blocked by pretreatment with inhibitors of the MAPK pathway [U0126 (100 μg/kg, i.p.), SB203580 (40 μg/ml, 10 ml/kg, i.p.), or SP600125 (50 μg/kg, i.p.)], as measured by von Frey testing. **(H)** The extent and duration of analgesia are estimated by the area under curve (AUC (g min)) of PWT vs time (0-120 minutes). Data are expressed as the mean ± SEM. **p < 0.01 and ***p < 0.001 for sham group vs SNI group. #*p* < 0.05, and ##*p* < 0.01 for SNI group vs AG+SNI group. ^&&^*p* < 0.01 compared with AG+SNI group. The "ns" indicates no significant difference.

AG has been reported to regulate a variety of physiological and pathological functions through the activation of the MAPK (p38, ERK and JNK), mTOR, and AMPK, pathway (12, 13, 20). Additionally, activation of these signal pathways can mitigate neuropathic pain in a wide range of preclinical pain models (52–54), and represents a promising therapeutic target for the modulation of chronic pain (52). Accordingly, we next assessed the role of the MAPK, mTOR, PGC-α and AMPK, signaling pathways in the antiallodynic effects of AG. As shown in **Figures 8A–C**, the western blot assays showed that SNI-induced higher levels of phosphorylated of ERK, JNK and p38 in the ipsilateral dorsal horn (*p* < 0.01 for p-ERK, *p* < 0.001 for p-JNK, *p* = 0.078 for p-p38, **Figures 8A–C**). Whereas AG treatment significantly inhibited ERK, JNK and p38 phosphorylation in L4-L5 spinal segments (*p* < 0.01 for p-ERK and p-JNK, *p* < 0.05 for p-p38, **Figures 8A–C**). Meanwhile, as shown in **Figures 8D–F**, the expression levels of PGC-α and the phosphorylated of mTOR and AMPK were not affected by AG treatment. These data indicate that the analgesic effect of AG is associated with reduced phosphorylation of MAPK.

Subsequently, to further validate the critical role of the MAPK pathway in AG’s effects, we employed the ERK inhibitor U0126, p38 inhibitor SB20358 and JNK inhibitor SP600125 to block AG-induced responses. We found that pretreatment with the U0126 (100 μg/kg, i.p.), or SB203580 (40 μg/ml, 10 ml/kg, i.p.), or SP600125 (50 μg/kg, i.p.), 30 min prior to AG administration (25 mg/kg; i.p.), blocked the analgesic effects of AG in SNI mice (*p* < 0.001 for SNI vs SNI + AG, *p* < 0.01 for SNI + AG vs SNI + AG + U0126/SB203580/SP600125) (**Figures 8G, H**). In summary, pharmacological inhibition of MAPK attenuated the analgesic effect of AG, suggesting a potential role of this pathway in its mechanism.

### 3.7 AG’s broad-spectrum analgesic activity

In addition to neuropathic pain, we also investigated the effects of AG in various pain models, including formalin-induced acute inflammatory pain, CFA-induced chronic inflammatory pain, capsaicin-induced hyperalgesia, and acetic acid-induced visceral pain. The results demonstrated that AG exhibits significant analgesic effects across these diverse models, indicating its broad-spectrum analgesic properties. The specific findings are detailed below.

In the formalin test, consistent with previous reports, significant flinching behaviors were evoked by formalin in both phase I and II (31). Phase І represented acute pain and phases II represented acute inflammatory pain. The baseline values (time spent on biting injection paw) obtained from saline treated mice were 176.3 ± 11.2 s in phase І and 246.9 ± 31.7 s in phase ІІ. Compared with the saline group, treatment of mice with AG (5, 10, 25 and 50 mg/kg, i.p.) dose-dependently decreased the time spent on licking in phase І (*p* < 0.01 for 10, 25 and 50 mg/kg AG vs saline group) (**Supplementary Figure S2B**). In phase ІІ, we observed that i.p. administration of AG (5, 10, 25 and 50 mg/kg) decreased the nociceptive responses to formalin (*p* < 0.01 for 10 and 50 mg/kg AG vs saline group, *p* < 0.05 for 25 mg/kg AG vs saline group, **Supplementary Figure S2C**). Meanwhile, morphine (10 mg/kg) in phase І and ІІ significantly induced antinociceptive effects (*p* < 0.01 for phase І and phase ІІ).

The duration of capsaicin-evoked acute pain (nocifensive behaviors), consisting of paw licking and flinching, were calculated in 15 min after injection. Capsaicin-induced secondary mechanical hyperalgesia in the injected area, were calculated in 20-30 and 45-60 min after injection. As shown in **Supplementary Figure S3**, capsaicin in saline mice showed a significant reduction in mechanical PWT at 30 and 60 min after injection (**Supplementary Figure S3A**). Pretreatment with AG (10, 25 and 50 mg/kg, i.p.) dose-dependently attenuated mechanical allodynia (*p* < 0.05 for 10 mg/kg AG or morphine group, *p* < 0.01 for 25 mg/kg AG or 50 mg/kg AG group, **Supplementary Figures S3B, C**). Moreover, capsaicin in saline mice evoked a nocifensive behavior that was maximal in both the licking duration (**Supplementary Figure S3D**) and the frequency (**Supplementary Figure S3E**) during the first 15-minute post-injection period. AG treatment reduced a dose-related the total nocifensive behavior including the licking duration and frequency (*p* < 0.05, **Supplementary Figures S3D, E**). These results indicate that i.p. injection of AG significantly inhibited capsaicin-evoked acute pain.

To assess the antinociceptive effects of AG in chronic inflammatory pain, we established the chronic inflammatory pain model in mice with intraplantar (i.pl.) injection of CFA, and mechanical hyperalgesia was tested before and 1h, 2h, 4h and 24h after i.pl. injection of CFA. As shown in **Supplementary Figure S4**, CFA treated mice exhibited mechanical hyperalgesia (0.206 ± 0.08 g) at 3 days post-injection (**Supplementary Figure S4B**). AG (5, 10, 25 and 50 mg/kg) were i.p. injected at the time points of 3 d after CFA injection in mice. The results showed that i.p. administration of AG (5, 10, 25 and 50 mg/kg) significantly and reversibly mitigated mechanical allodynia (*p* < 0.01 for 5 mg/kg CFA + AG vs CFA group, *p* < 0.001 for 10, 25 and 50 mg/kg AG + CFA vs CFA group) (**Supplementary Figures S4B, C**). Similarly, i.p. administration of AG (5, 25 and 50 mg/kg) at 3 d after CFA injection reversed thermal hyperalgesia (*p* < 0.01 for 25 mg/kg CFA + AG vs CFA group, *p* < 0.001 for 50 mg/kg AG + CFA vs CFA group, **Supplementary Figures S4D, E**). These data demonstrate that AG can produce immediate mitigation in mechanical allodynia and thermal hyperalgesia in CFA induced chronic inflammatory pain state.

The analgesic actions of i.p. AG were further investigated in the acetic acid-induced writhing test. Acetic acid evoked significantly writhe behaviors and the baseline value (number of writhes) obtained from saline treated mice was 49.5 ± 1.5. Subsequently, i.p. pretreatment with AG (10, 25 and 50 mg/kg) significantly inhibited abdominal constrictions induced by acetic acid in a dose-related manner (*p* < 0.05 for 10 mg/kg AG vs acetic acid group, *p* < 0.01 for 25 mg/kg or 50 mg/kg AG vs acetic acid group) (**Supplementary Figure S5B**). These data suggested that AG significantly produced the antiallodynic effects in the acetic acid-induced writhing model.

### 3.8 Evaluation of the short-term toxicity effects of AG

**Figure 9:**
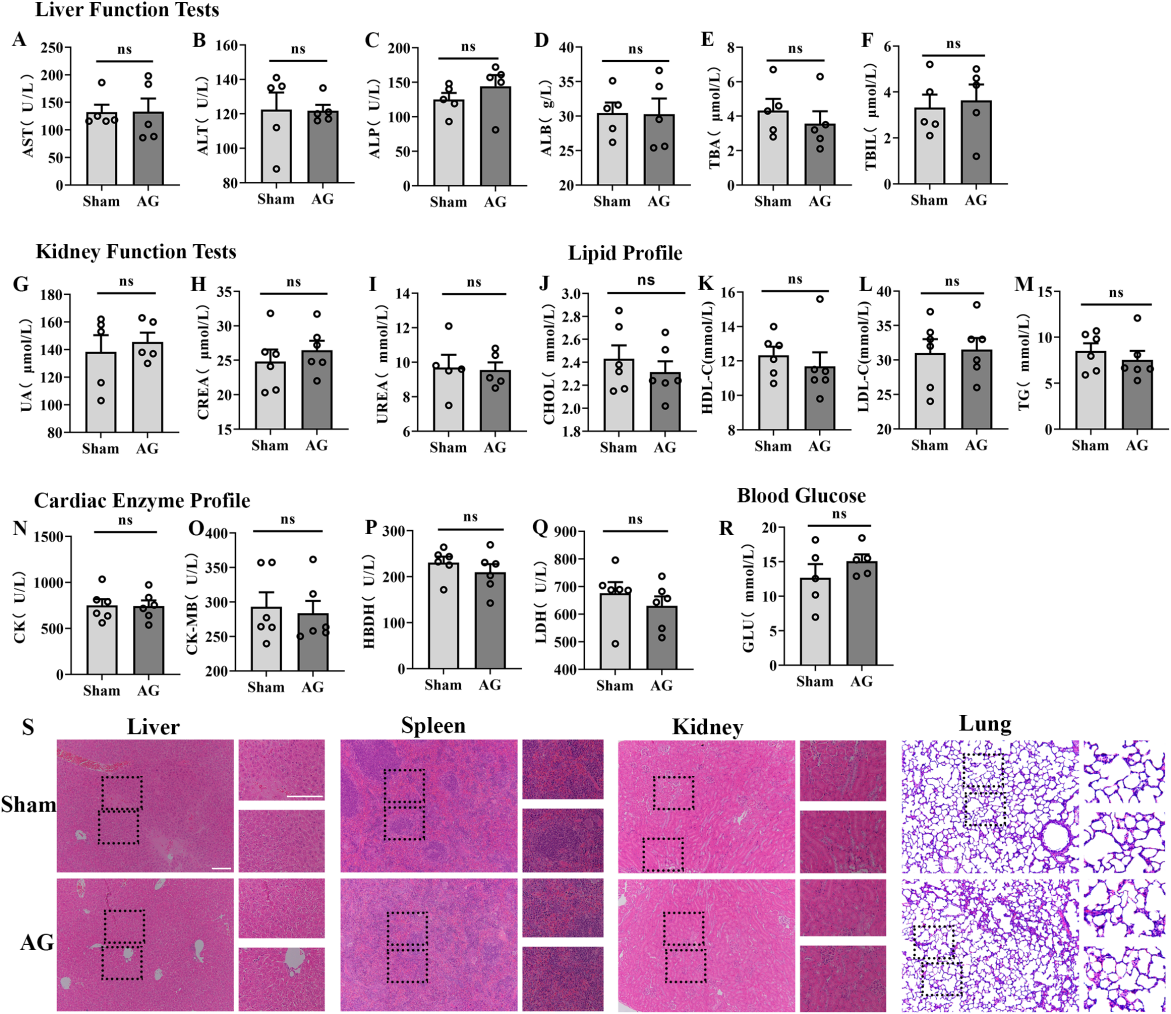
The toxicity effects of chronic injection of AG in plasma and pathological situations. **(A–F)** Qualitative data collected from plasma of mice in different groups referred to liver function (n = 5). **(G–I)** Qualitative data collected from plasma of mice in different groups referred to kidney function (n = 5). **(J–M)** Qualitative data collected from plasma of mice in different groups referred to lipid function (n = 5). **(N–Q)** Qualitative data collected from plasma of mice in different groups referred to cardiac enzyme function (n = 5). **(R)** Qualitative data collected from plasma of mice in different groups referred to blood glucose (n = 5). **(S)** The effects of AG (50 mg/kg) in liver, spleen, kidney and lung pathological situations by H&E staining analyses. Data are expressed as the Mean ± SEM. The "ns" indicates no significant difference.

In the acute toxicity experiment, we investigated the effects of intraperitoneal injection of AG for one consecutive week on the short-term biochemical screening, including liver function, renal function, lipid function, myocardial enzyme profile and blood glucose (**Figures 9A–R**), and the histopathological examination, including histological damage of liver, spleen, kidney and lung (**Figure 9S**). As shown in **Figures 9A–R**, ELISA data showed intraperitoneal injection of AG (50 mg/kg) for one consecutive week did not alter the levels of liver function factors (AST, ALT, ALP, ALB, TBA, and TBIL, **Figures 9A–F**), kidney function factors (UA, CREA, and UREA, **Figures 9G–I**), lipid profile factors (CHOL, HDL-C, LDL-C, and TG, **Figures 9J–M**), cardiac enzyme profile factors (CK, CK-MB, HBDH, and LDH, **Figures 9N–Q**) and glucose in plasma (**Figure 9R**). Meanwhile, in H&E staining analyses, mice intraperitoneally administered high-dose AG (50 mg/kg) for one consecutive week did not produce significant effects in liver, spleen, kidney and lung pathological situations (**Figure 9S**). Collectively, short-term toxicity experiments show that chronic administration of AG has barely any effects on liver, renal, lipid, and cardiac function in mice.

### 3.9 Evaluation of the side effects of AG

**Figure 10:**
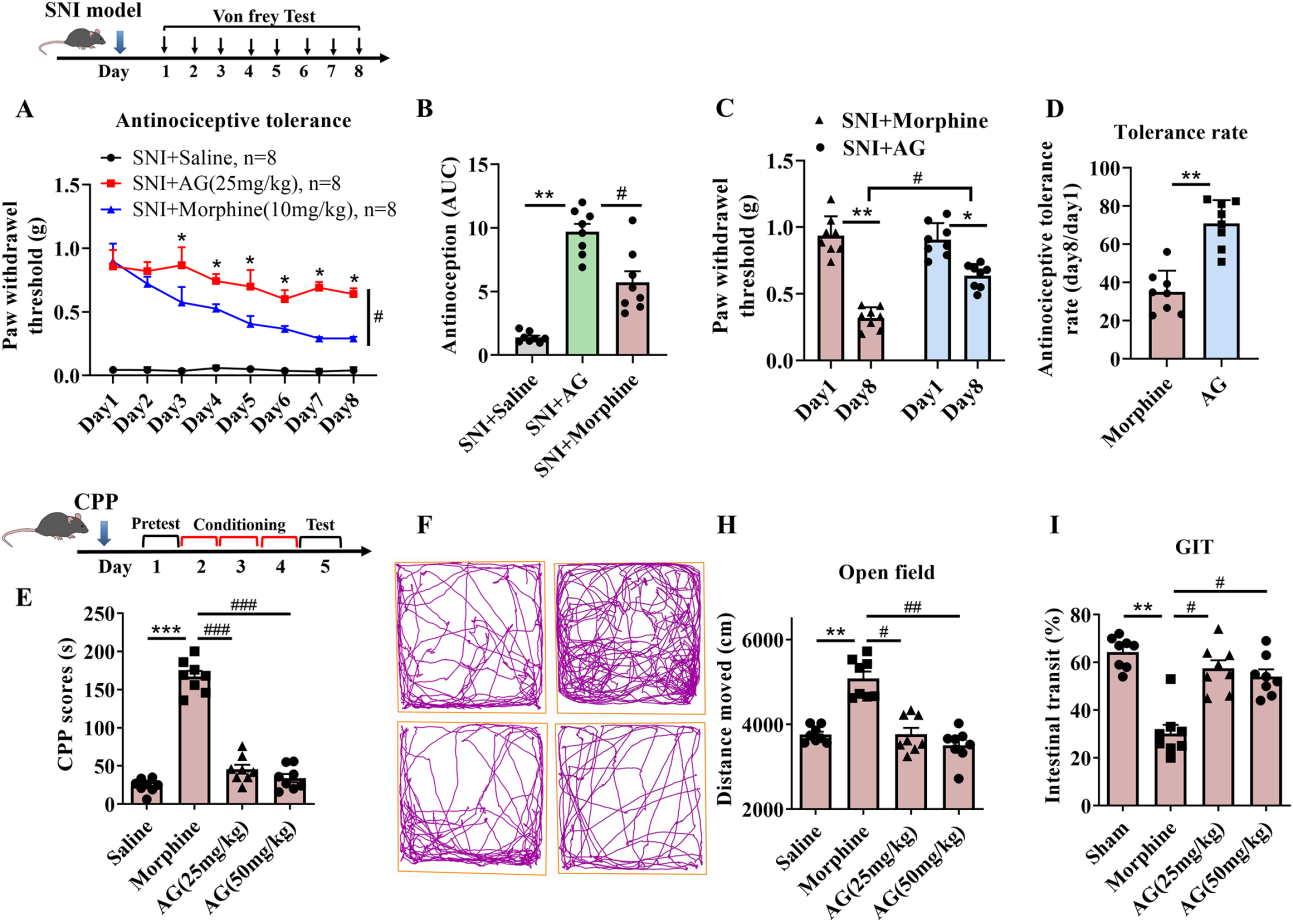
Evaluation of the side effects of AG. **(A–D)** Effects of the repeated administration of AG and morphine were investigated on antinociceptive tolerance in SNI induced neuropathic pain model. **(B)** AUC for AG and morphine at Day 1- Day 8 after repeated i.p. administration. **(C)** Antinociceptive effects of AG and morphine at Day 1 and Day 8. **(D)** Antinociceptive tolerance rate (Day 8/Day 1) of AG and morphine. **(E)** The addictive effects of AG and morphine were investigated in the CPP test. **(F–H)** Effects of i.p. administration of AG and morphine on locomotor activity in the open field test. **(I)** The effects of i.p. administration of AG and morphine on gastrointestinal transit in mice. Results are expressed as the Mean ± SEM. n = 8-10 mice per group. **p < 0.01 and ***p < 0.001 compared with saline group. ^#^*p* < 0.05, ^##^*p* < 0.01 and ^###^*p* < 0.001 compared with morphine group.

Repeated administration of opioids and non-steroidal anti-inflammatory drugs (NSAIDs), the most widely used analgesics for pain management, is known to cause adverse effects such as tolerance, addiction, hyperalgesia, and constipation (55–57). To evaluate the development of antinociceptive tolerance to AG in SNI mice, we administered AG (25 mg/kg, i.p.) or morphine (10 mg/kg, i.p.) once daily for eight consecutive days and assessed their effects on mechanical hyperalgesia within 0-120 minutes after each daily injection (**Figures 10A, B**). After eight days of repeated administration, the analgesic effect of AG slightly decreased but retained 73% of its efficacy (*p* = 0.4541, **Figures 10C, D**), whereas morphine exhibited a marked loss of efficacy, retaining only 37% by day 8 (*p* < 0.01, **Figures 10C, D**). These results indicate that intraperitoneal administration of AG significantly attenuates the development of analgesic tolerance compared to conventional analgesics.

For the addiction experiments, morphine was used as the positive control, representing a traditional opioid analgesic. In the CPP test, neither the 25 mg/kg nor 50 mg/kg dose of AG induced any addictive effects. In contrast, morphine administration resulted in a significant place preference, indicating marked addictive potential (*p* < 0.001, **Figure 10E**).

Motor function is not only a notable side effect of opioid analgesics but also a critical factor that can lead to false-positive outcomes in the von Frey test. Therefore, we evaluated the impact of AG on spontaneous locomotor activity using the open field test (*p* < 0.01, **Figures 10F–H**). As shown in **Figure 10H**, morphine significantly increased locomotor activity (*p* < 0.01, **Figure 10H**). Notably, AG at both 25 mg/kg and 50 mg/kg did not produce any significant effect on spontaneous movement.

Given that opioid analgesics such as morphine are known to inhibit GI transit and NSAIDs like celecoxib can induce GI toxicity (57), we next evaluated the potential impact of AG on GI motility. Intraperitoneal administration of AG at 25 or 50 mg/kg resulted in a modest, non-significant reduction in gastrointestinal transit compared with control animals (GI transit rate [GIT%]: control, 64.4 ± 5. 2; AG 25 mg/kg, 58.3 ± 6.1; AG 50 mg/kg, 56.4 ± 3.4). In contrast, morphine (10 mg/kg, i.p.) significantly inhibited GI transit (GIT%: 32.2 ± 3.3, *F_3,34_* = 14.53, *p* < 0.01, **Figure 10I**).

Based on the comprehensive assessment of AG’s side effect profile, the compound demonstrates minimal antinociceptive tolerance and negligible gastrointestinal transit inhibition, while exhibiting no adverse effects on locomotor function under the experimental conditions. These findings collectively suggest that AG represents a promising therapeutic candidate with potent analgesic efficacy and a favorable safety profile for neuropathic pain management.

## 4 Discussion

Neuropathic pain (NP) remains a persistent medical challenge that severely impacts human quality of life (4, 58). Current pharmacological treatments are still the primary approach for pain relief, and although significant progress has been made in NP drug research in recent years, many patients continue to exhibit poor responses to existing therapies (1, 4). Consequently, the identification of novel drug targets is urgently needed and holds critical importance for advancing neuropathic pain medication development. AG, a lignan compound derived from plants such as *Arctium lappa* (burdock), has attracted considerable attention in recent years due to its remarkable antioxidant and anti-inflammatory properties (12, 13, 15, 59). This study revealed the unique and synergistic therapeutic advantages of AG as a treatment strategy for neuropathic pain animal models.

To evaluate the potential role of AG in neuropathic pain, we established a spared nerve injury (SNI)-induced neuropathic pain mouse model. We found that SNI surgery elicited significant mechanical allodynia and thermal hyperalgesia one week post-operation. However, intraperitoneal administration of AG robustly alleviated SNI-induced neuropathic pain in a dose-dependent manner, underscoring its potential pharmacological effects as a therapeutic agent against this condition. Meanwhile, we observed that AG treatment exerted potent inhibitory effects on formalin-induced flinching behaviors in both Phase I and Phase II, suggesting that AG inhibits acute inflammatory pain induced by formalin. Subsequently, we assessed the effects of AG on chronic inflammatory pain. Previous studies have documented that chronic inflammatory pain arises from chemical stimuli, tissue damage, or autoimmune processes (60, 61). Complete Freund’s Adjuvant (CFA) is widely used to establish chronic inflammatory pain models (61). In this study, we found that CFA injection triggered mechanical hyperalgesia 3 days post-injection, whereas AG administration significantly attenuated CFA-induced mechanical hyperalgesia and thermal pain sensitivity. In addition to formalin-induced acute inflammatory pain and CFA-induced chronic inflammatory pain, we also investigated the effects of AG in various pain models, including capsaicin-induced hyperalgesia and acetic acid-induced visceral pain. The results demonstrated that AG exhibits significant analgesic effects across these diverse models, indicating its broad-spectrum analgesic properties.

To further investigate the mechanisms underlying AG’s regulation of neuropathic pain, we performed transcriptome sequencing on spinal cord tissues. The results revealed that AG treatment modulated signaling pathways associated with inflammatory responses, immune cells, oxidative stress, and mitochondrial biogenesis. Growing evidence has illustrated that oxidative stress serves as a stimulus for inflammatory responses, inducing the production of various genes, including inflammatory factors and cytokines (21, 22, 25). Furthermore, excessive pro-inflammatory cytokines can act as ROS-activating factors, triggering oxygen radical production (26). Astrocytes and microglia have been reported to play critical roles in the development of chronic neuropathic pain (24). In our study, AG significantly suppressed microglial and astrocyte activation, as well as pro-inflammatory cytokine production in the spinal cord. These findings align with previous studies demonstrating AG’s anti-inflammatory properties (13, 18–20). For instance, AG alleviated joint swelling and inflammatory infiltration in a rat arthritis model (19) and reduced IL-6 and TNF-α levels in LPS-induced macrophages (18).

Next, we explored whether mitochondrial function and oxidative damage were involved in AG’s effects in the SNI model. qPCR and ELISA analyses demonstrated that AG treatment significantly enhanced the production of antioxidant factors (NQO1, GSH, and SOD) while inhibiting oxidative damage markers (CYP2E1, BAX, MDA, and ROS) in SNI-induced animals. These results are consistent with existing literature on AG’s roles in oxidative stress and mitochondrial protection (12, 62). For example, AG ameliorated oxidative stress via the Nrf2-dependent pathway in a diabetic mouse model (14), improved neuronal survival and reduced ROS levels in H_2_O_2_-induced oxidative injury (17), and preserved mitochondrial function in dopaminergic neurons in a Parkinson’s disease model (10). Collectively, these data suggest that AG’s analgesic effects in neuropathic pain may stem from its dual inhibition of inflammatory responses and oxidative stress, coupled with mitochondrial protection.

In addition to assessing factors associated with oxidative damage, we measured 8-hydroxy-2’-deoxyguanosine (8-OHdG), an oxidative DNA nucleoside modification widely recognized as a biomarker of ROS generation and DNA damage in both nuclear and mitochondrial genomes, as reported in previous studies (48, 49). We observed that SNI simulation increased expression of spinal 8-OHdG, whereas AG application had a negative effect on 8-OHdG expression. Transmission electron microscopy analysis further revealed ultrastructural mitochondrial abnormalities in SNI mice, characterized by cristae disintegration and reductions in both mitochondrial size and perimeter. Notably, AG-treated animals exhibited mitochondrial preservation comparable to sham-operated controls, maintaining intact double-membrane structures with well-organized oval-shaped cristae and typical organelle morphology.

To identify cell types associated with mitochondrial oxidative damage in the spinal dorsal horn, we performed dual immunofluorescence staining of 8-OHdG with neuronal (NeuN) and glial markers (Iba1 for microglia and GFAP for astrocytes). Quantitative analysis revealed predominant co-localization of 8-OHdG signals with neuronal nuclei (NeuN+ cells), with minimal overlap observed in microglial (Iba1+) or astrocytic (GFAP+) populations. This cell-type-specific oxidative damage pattern suggests that AG’s therapeutic effects in SNI may primarily involve neuroprotective mechanisms within spinal neurons.

Subsequently, we investigated neuronal activation patterns in the spinal dorsal horn, focusing on nociceptive sensory pathways. c-Fos protein expression, a well-established marker of neuronal activation, was quantified alongside calcitonin gene-related peptide (CGRP), which labels activated nociceptive sensory neurons in chronic pain states (63, 64). Immunohistochemical analysis demonstrated that AG treatment significantly attenuated SNI-induced elevations in both c-Fos+ nuclei density and CGRP+ neuronal projections within the dorsal horn, indicating suppression of pain-related neuronal hyperactivity. Collectively, these findings demonstrate that AG exerts dual protective effects in SNI pathophysiology: 1) mitigating ROS-mediated oxidative neuronal damage through 8-OHdG reduction, and 2) attenuating maladaptive neuronal activation in spinal nociceptive processing pathways. Next, we further verified whether the inhibition of spinal microglia activation is a key factor in the analgesic effect of AG using minocycline, an inhibitor of microglial and astrocyte activation (65). We found that minocycline reduced but did not completely block the analgesic effect of AG, indicate that the inhibition of spinal dorsal horn glial cell activation is involved in the analgesic effect of AG, but it is not an essential, obligatory step.

To elucidate the molecular mechanism underlying AG’s modulation of neuropathic pain, we systematically investigated key pathways (MAPK/AMPK/PGC-1α/mTOR signaling axis), which coordinates mitochondrial biogenesis and nociceptive processing in chronic pain states. Mechanistically, SNI induced significant upregulation of PGC-1α protein expression coupled with hyperphosphorylation of MAPK family members: ERK, JNK, and p38, along with mTOR activation. AG treatment specifically normalized MAPK overactivation, reducing phospho-ERK, phospho-JNK, and phospho-p38 levels, but does not affect the expression of p-mTOR, p-AMPK_1/2_, and PGC-1α expression. A reduction in MAPK phosphorylation was correlated with the analgesic outcome following AG administration. To establish causal relationships, we employed pharmacological inhibition using U0126 (ERK inhibitor), SB203580 (p38 inhibitor), and SP600125 (JNK inhibitor) administered 30 min prior to AG treatment. Intriguingly, all three MAPK inhibitors completely abolished AG’s analgesic efficacy in mechanical allodynia tests. Taken together, the attenuation of AG’s analgesic effect upon MAPK inhibition points to a possible mechanistic involvement of this pathway.

In addition, for analgesic drugs, the analgesic effect and mechanism are very important, but drug toxicity and side effects are even more critical. Therefore, we proceeded to evaluate the acute short-term toxicity and analgesic side effects of AG. To assess whether short-term AG administration induces significant toxicity, we intraperitoneally injected a high dose of AG daily for one week and performed biochemical screening and histological analyses on serum and tissues. Results revealed that chronic AG administration did not alter hepatic or renal function parameters, glucose and lipid metabolism-related markers, or myocardial enzyme profiles. Furthermore, no obvious tissue damage or inflammatory responses were observed in the liver, kidneys, spleen, or lungs. These findings suggest that AG may show no significant toxicity in short-term experiments; however, for pre-clinical drug target research, short-term toxicity data are far from sufficient to establish the safety profile of AG. Further comprehensive toxicological studies are necessary, including chronic dosing, histopathological examination, and assessment of major organ systems, before any definitive conclusions regarding safety can be drawn.

Traditional analgesics such as morphine often induce severe side effects in clinical applications, including analgesic tolerance, constipation, addiction, and locomotor hyperexcitability. Therefore, we next evaluated the effects of AG on analgesic tolerance, addiction, spontaneous locomotor activity, and constipation. The results showed that the compound demonstrates minimal antinociceptive tolerance and negligible gastrointestinal transit inhibition, while exhibiting no adverse effects on locomotor function under the experimental conditions. It is important to note that morphine was used herein as a pharmacological tool to benchmark potency rather than as a clinical recommendation. Future studies are essential to directly compare the efficacy and safety profile of AG with first-line neuropathic pain agents, such as pregabalin or gabapentin, in relevant models.

## 5 Conclusion

In summary, these findings collectively demonstrate that AG was able to attenuate neuropathic pain in SNI-induced mechanical allodynia and thermal hyperalgesia at least partially through activation of MAPK signaling pathway. Mechanistic studies indicated that AG significantly restored mitochondrial oxidative damage, inhibited microglia and astrocyte activation, and decreased the production of pro-inflammatory factors, which contributed to pain relief. Furthermore, short-term toxicity and side effect studies showed that short-term administration of AG did not affect liver or kidney function, nor did it induce significant analgesic side effects commonly associated with traditional analgesics, such as tolerance, addiction, or constipation.

## Data Availability

The datasets presented in this study can be found in online repositories. The names of the repository/repositories and accession number(s) can be found in the article/**Supplementary Material**.
