# Supplementary material for: Targeting the oxidative stress-neuroinflammation axis: the mechanism of arctigenin’s broad-spectrum analgesia with limited side effects
Source: Front Immunol. 2026 Mar 5;17:1754756. doi: 10.3389/fimmu.2026.1754756 (PMC13000350; doi:10.3389/fimmu.2026.1754756)
Supplement: Supplementary file 1 [file DataSheet1.docx]

**Supplementary material**

**Targeting the Oxidative Stress-Neuroinflammation Axis: The Mechanism of Arctigenin's Broad-Spectrum Analgesia with Limited Side Effects**

Zhe Wang^1*^, Shu Li^2,3*^, Ping Lu^2^, Jinglei Liao^2^, Yimin Xu^2^, Chen Lu^2#^, Weiwei Li^4#^, Jinhong Jiang^#2^

1. School of Food and Biological Engineering, Xuzhou University of Technology, Xuzhou 221018, China
2. Jiangsu Province Key Laboratory of Anesthesiology, Jiangsu Province Key Laboratory of Anesthesia and Analgesia Application Technology, NMPA Key Laboratory for Research and Evaluation of Narcotic and Psychotropic Drugs, Xuzhou Medical University, 209 Tongshan Rd, Xuzhou, Jiangsu 221004, China.
3. Pharmacy College of Shihezi University/Key Laborataty of Xinjiang Phytomedicine Resource and Utilization, Ministry of Education/ Collaborative Innovation Center for Efficient Safflower Production and Resource Utilization of XPCC/ Institute for Safflower Industry Research，Shihezi University, Shihezi.
4. Department of Anesthesiology, Xuzhou Maternal and Children Health Care Hospital, Xuzhou, China

*The authors equally contributed to this work

**#Corresponding author:** E-mail addresses: [100002019018@xzhmu.edu.cn](mailto:100002019018@xzhmu.edu.cn) (**Jinhong Jiang**), [leevee@126.com](mailto:leevee@126.com) (Weiwei Li), [lucoct@163.com](mailto:lucoct@163.com) (Chen Lu)

**Fig.S1.** AG attenuates SNI-induced mechanical pain hypersensitivity in female. (A)The anti-hyperalgesic effects of AG (10, 25, and 50 mg/kg) in SNI female mice. (B) The AUC values in female. (C) A comparison of the effects of AG on mechanical pain in female and male. Data are expressed as the Mean ± SEM. n = 8 mice per group. ***p* < 0.01 and ****p* < 0.001 compared with SNI+saline group.

**
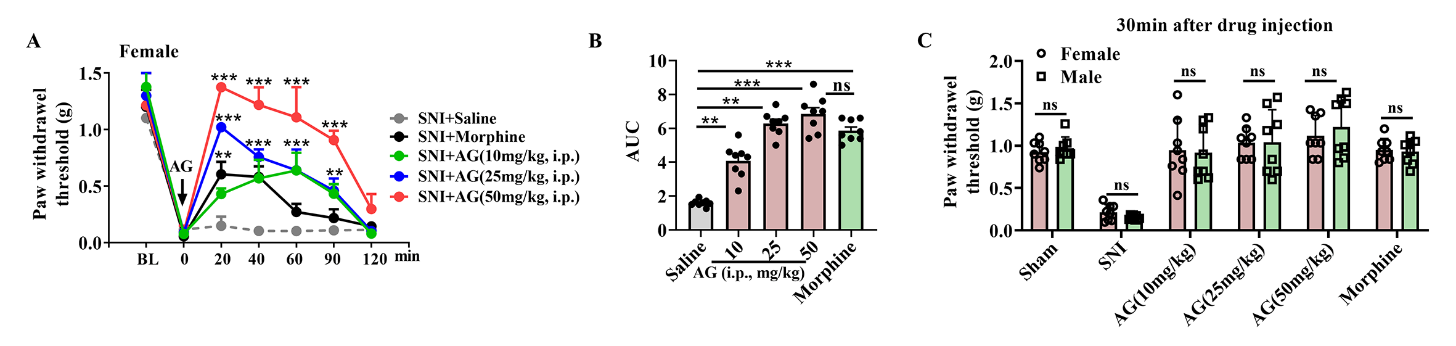
**

**Fig.S2.** Antinociceptive effects of AG in formalin pain models. (A) Experimental design. (B, C) Antinociceptive effects of AG in the mouse formalin test. n = 8-10. Data are expressed as the mean ± SEM. **p*<0.05, and ***p*<0.01 compared with saline+formalin group.


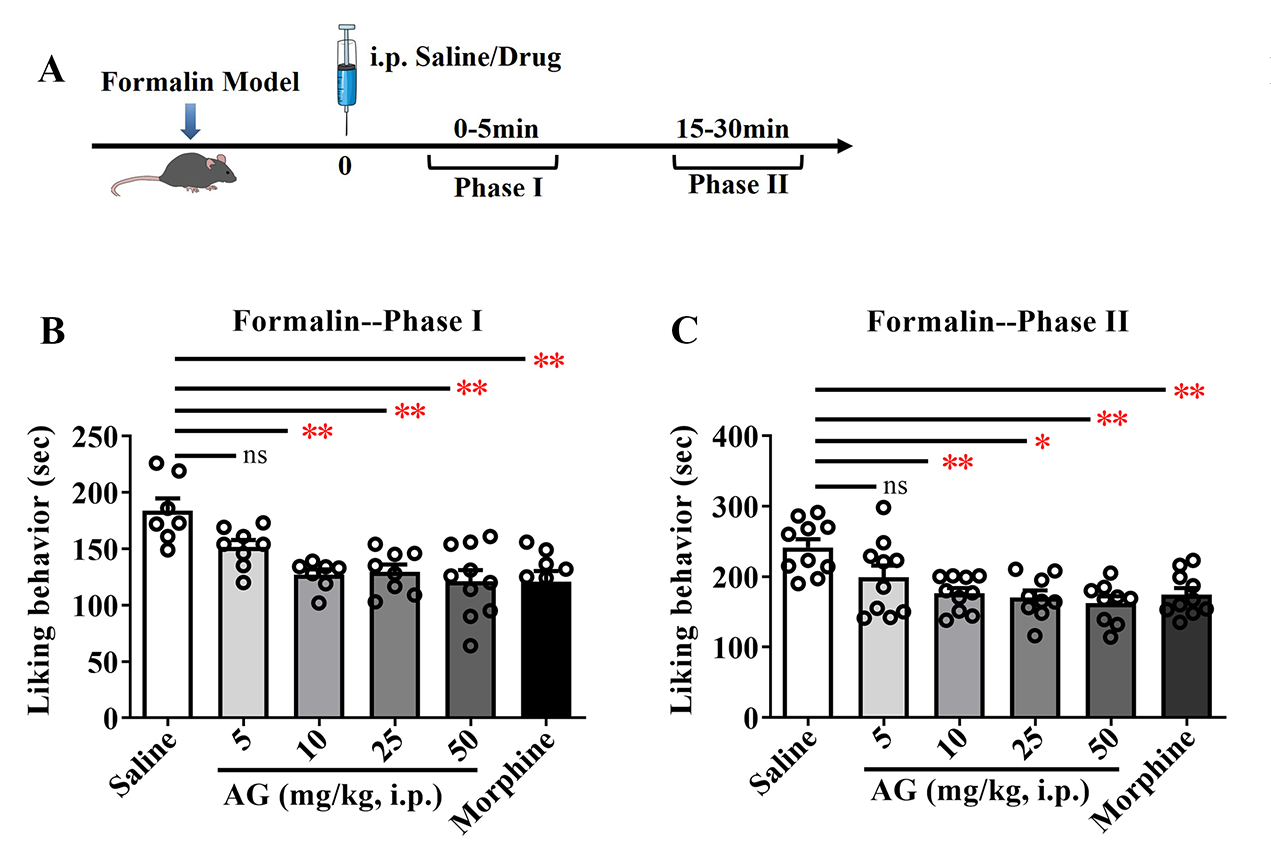


**Fig.S3.** Antinociceptive effects of AG in capsaicin-induced hyperalgesia model. (A) Experimental design. (B) i.p. injection of AG mitigated the capsaicin-induced reduction of the mechanical pain threshold. (C) The extent and duration of analgesia are estimated by the area under curve (AUC (g/min)) of PWT vs time (0-60 minutes). (D-E) i.p. injection of AG suppressed capsaicin-induced acute nocifensive behaviors (such as hind-paw licking and flinching). n = 8-10. Data are expressed as the mean ± SEM. **p*<0.05, and ***p*<0.01 compared with saline+capsaicin group. ^#^*p*<0.05, and ^##^*p*<0.01 for morphine group vs AG group.


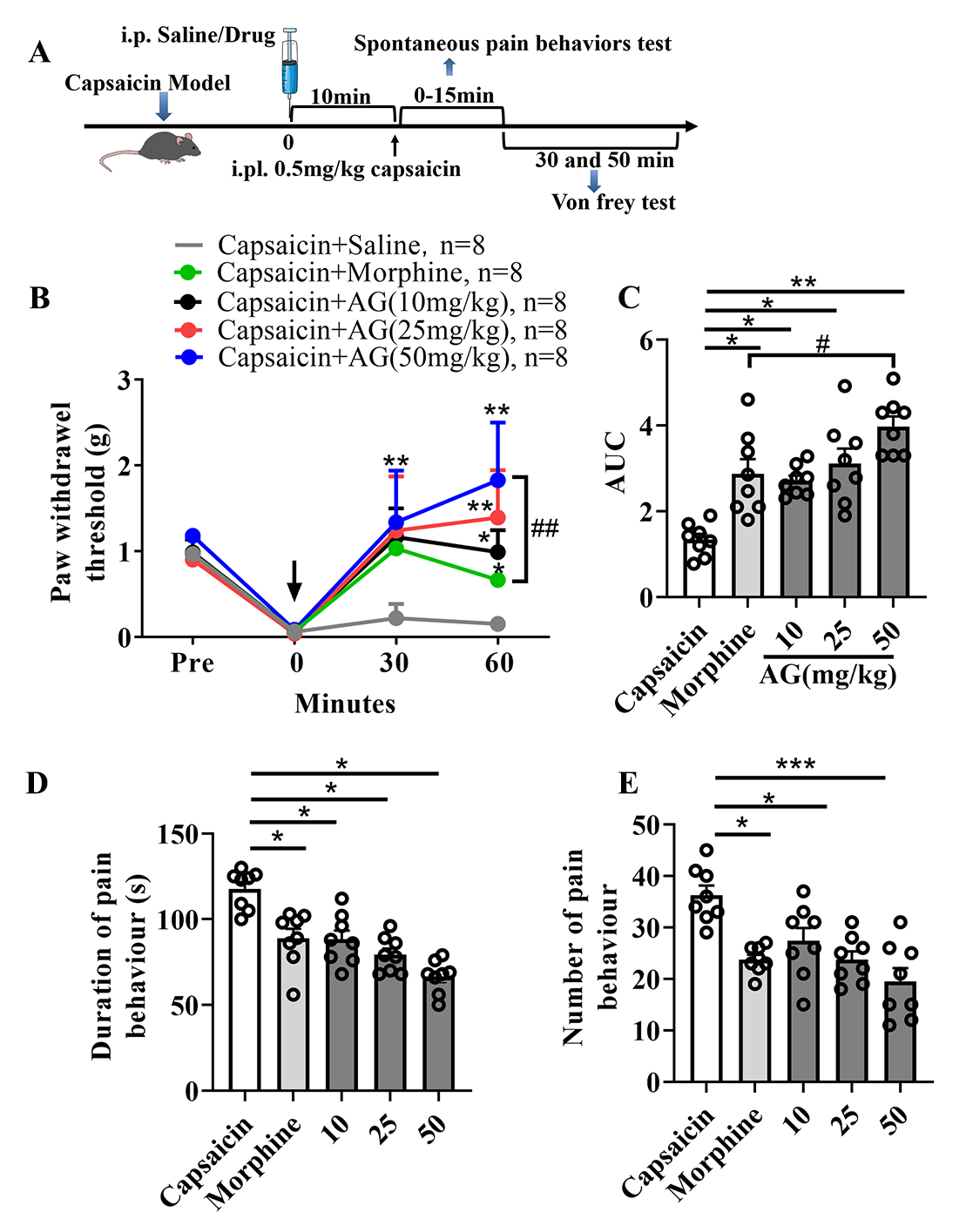


**Fig.S4.** Antinociceptive effects of AG in CFA pain models. (A) Experimental design (B, D) Antinociceptive dose- and time-response curve of AG after i.p. administration. n = 6. (C, E) The extent and duration of analgesia are estimated by the area under curve (AUC (g/min)) of PWT vs time (0-120 minutes). n = 8-10. Data are expressed as the mean ± SEM. **p*<0.05, ***p*<0.01 and ****p*<0.01 compared with saline+CFA group. ^&^*p*<0.05, and ^&&^*p*<0.01 for morphine group vs AG group.


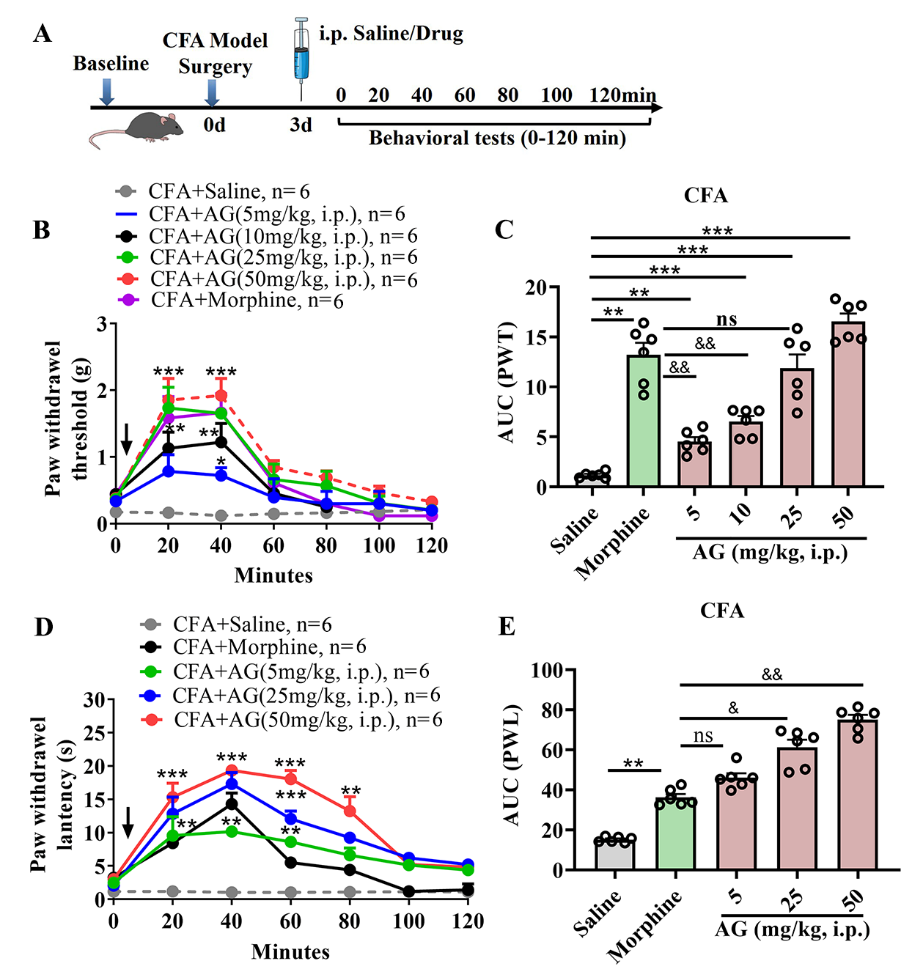


**Fig.S5.** Antinociceptive effects of AG in acetic acid-induced visceral pain models. (A) Experimental design (B) Antinociceptive effects of the i.p. injection of AG in the mouse acetic acid writhing test. n = 8-10. Data are expressed as the mean ± SEM. **p*<0.05, and ***p*<0.01 compared with saline+acetic acid group.


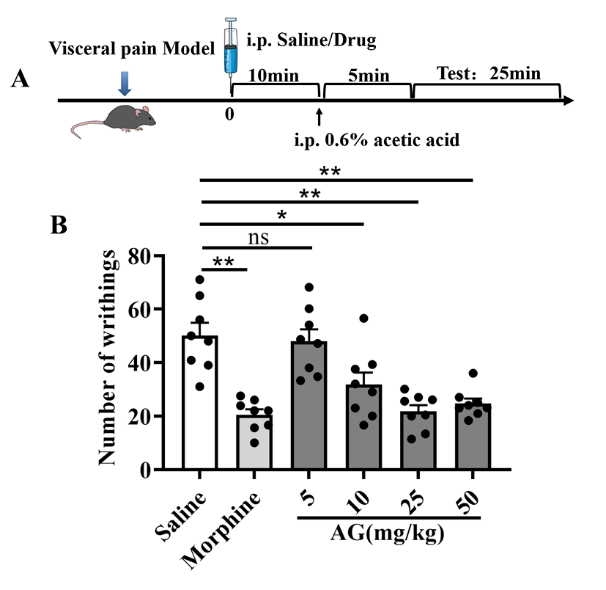


Table S1: Antibody information used in this experiment.

| Antibody Name | Dilution Concentration | Cat Number | Company |
| --- | --- | --- | --- |
| Anti-p-AMPK rabbit mAb | 1:1000 | 5831S | CST, USA |
| Anti-p-mTOR rabbit mAb | 1:1000 | 5536 | CST, USA |
| Anti-PGC-1α mouse mAb | 1:5000 | 66369-1-lg | Proteintech, China |
| Anti-p-ERK rabbit mAb | 1:1000 | 4370 | CST, USA |
| Anti-t-ERK rabbit mAb | 1:1000 | 4695 | CST, USA |
| Anti-p-JNK mouse mAb | 1:1000 | 4668 | CST, USA |
| Anti-t-JNK rabbit mAb | 1:1000 | 9252 | CST, USA |
| Anti-p-p38 rabbit mAb | 1:1000 | 4511 | CST, USA |
| Anti-t-p38 rabbit mAb | 1:1000 | 8690 | CST, USA |
| Anti-CGRP rabbit mAb | 1:1000 | 14959 | CST, USA |
| Anti-c-fos rabbit mAb | 1:2000 | 2250S | CST, USA |
| Anti-8-OHdG mouse mAb | 1:500 | ab62623 | Abcam, USA |
| Anti-NeuN rabbit mAb | 1:500 | ab177487 | Abcam, USA |
| Anti-iba1 rabbit mAb | 1:500 | 019-19741 | Wako, Japan |
| Anti-GFAP rabbit mAb | 1:500 | ab33922 | Abcam, USA |
| Anti-GAPDH rabbit mAb | 1:1000 | GB12002 | Servicebio, China |

Table S2: Primers information used in this experiment.

| Gene | Prime | Sequence（5’-3’） |
| --- | --- | --- |
| *cyp2e1* | sense | CTTAGGGAAAACCTCCGCAC |
|  | anti-sense | GGGACATTCCTGTGTTCCAG |
| *bax* | sense | GATCAGCTCGGGCACTTTAG |
|  | anti-sense | TTGCTGATGGCAACTTCAAC |
| *sod1* | sense | AACCAGTTGTGTTGTCAGGAC |
|  | anti-sense | CCACCATGTTTCTTAGAGTGAGG |
| *nqo1* | sense | AGCGTTCGGTATTACGATCC |
|  | anti-sense | AGTACAATCAGGGCTCTTCTCG |
| *psmb8* | sense | CAGTCCTGAAGAGGCCTACG |
|  | anti-sense | CACTTTCACCCAACCGTCTT |
| *gbp2* | sense | GGGGTCACTGTCTGACCACT |
|  | anti-sense | GGGAAACCTGGGATGAGATT |
| *ggta1* | sense | CAGCCCTTCTTCCTGTTCAT |
|  | anti-sense | TGATGGGAGGTGTTGATGCT |
| *ligp1* | sense | GGGGCAATAGCTCATTGGTA |
|  | anti-sense | ACCTCGAAGACATCCCCTTT |
| *h2-d1* | sense | CTGGTGAGGAAGGAGATGGT |
|  | anti-sense | CAGCAAGTCAGGGTAGGACA |
| *tm4sfl* | sense | GCCCAAGCATCTTGTGGAGT |
|  | anti-sense | AGGGTAGGATGTGGCACAAG |
| *cd14* | sense | GGACTGATCTCATCCCCTCTG |
|  | anti-sense | GCTTCAGCCCAGTGAAAGAC |
| *slcloab* | sense | GCTTCGGTGGTATGATGCTT |
|  | anti-sense | CCACAGGCTTTTCTGGTGAT |
| *emp1* | sense | GAGACACTGGCCAGAAAAGC |
|  | anti-sense | TAAAAGGCAAGGGAATGCAC |
| *tgm1* | sense | AACCGGGAATATGAGTCCTCTG |
|  | anti-sense | CGTTGTTCTTAGTCACTTGGGC |
| *ptx3* | sense | AACAAGCTCTGTTGCCCATT |
|  | anti-sense | TCCCAAATGGAACATTGGAT |
| *il-1β* | sense | CAGCTTCAAATCTCGCAGCA |
|  | anti-sense | CTCATGTCCTCATCCTGGAAGG |
| *il-6* | sense | ACAACCACGGCCTTCCCTA |
|  | anti-sense | TCATTTCCACGATTTCCCAGA |
| *tnf-α* | sense | CATCTTCTCAAAATTCGAGTGACAA |
|  | anti-sense | CCAGCTGCTCCTCCACTTG |
| *il-18* | sense | GCCTCAAACCTTCCAAATCA |
|  | anti-sense | TGGATCCATTTCCTCAAAGG |
| *cd68* | sense | TGTCTGATCTTGCTAGGACCG |
|  | anti-sense | GAGAGTAACGGCCTTTTTGTGA |
| *il-4* | sense | GGTCTCAACCCCCAGCTAGT |
|  | anti-sense | GCCGATGATCTCTCTCAAGTGAT |
| *il-10* | sense | GCTCTTACTGACTGGCATGAG |
|  | anti-sense | CGCAGCTCTAGGAGCATGTG |
| *il-11* | sense | TGCTGACAAGGCTTCGAGTAG |
|  | anti-sense | ACATCAAGAGCTGTAAACGGC |
| *il-13* | sense | TGAGCAACATCACACAAGACC |
|  | anti-sense | GGCCTTGCGGTTACAGAGG |
| *gapdh* | sense | CGTCCCGTAGACAAAATGGT |
|  | anti-sense | TTGATGGCAACAATCTCCAC |

Table S3: Statistical analysis of the behavioral data used in this experiment were presented.

| Figure | Name | Number | Statistical analysis | p value |
| --- | --- | --- | --- | --- |
| Fig1A | SNI+saline | 8 | Two-way ANOVA followed by bonferroni post hoc tests | / |
|  | SNI+AG(5mg/kg) | 8 |  | ns |
|  | SNI+AG(10mg/kg) | 8 |  | 0.043 |
|  | SNI+AG(25mg/kg) | 8 |  | 0.0026 |
|  | SNI+AG(50mg/kg) | 8 |  | 0.0001 |
|  | SNI+morphine | 8 |  | 0.0088 |
| Fig1E | SNI+saline | 8 | Two-way ANOVA followed by bonferroni post hoc tests | / |
|  | SNI+AG(5mg/kg) | 8 |  | ns |
|  | SNI+AG(10mg/kg) | 8 |  | 0.031 |
|  | SNI+AG(25mg/kg) | 8 |  | 0.0076 |
|  | SNI+AG(50mg/kg) | 8 |  | 0.0001 |
|  | SNI+morphine | 8 |  | 0.0056 |
| Fig11A | SNI+saline | 8 | Two-way ANOVA followed by bonferroni post hoc tests | / |
|  | SNI+AG(25mg/kg) | 8 |  | 0.0001 |
|  | SNI+morphine(10mg/kg) | 8 |  | 0.0019 |
| Fig11B | SNI+saline | 8 | One-way ANOVA followed by bonferroni post hoc tests | / |
|  | SNI+AG(25mg/kg) | 8 |  | 0.0055 |
|  | SNI+morphine(10mg/kg) | 8 |  | 0.023 |
| Fig11D | SNI+AG(25mg/kg) | 8 | T test | / |
|  | SNI+morphine(10mg/kg) | 8 |  | 0.0073 |
| Fig11E | Saline | 8 | One-way ANOVA followed by bonferroni post hoc tests | / |
|  | Morphine | 8 |  | 0.0001 |
|  | AG(25mg/kg) | 8 |  | 0.0001 |
|  | AG(50mg/kg) | 8 |  | 0.0001 |
| Fig11H | Saline | 8 | One-way ANOVA followed by bonferroni post hoc tests | / |
|  | Morphine | 8 |  | 0.0042 |
|  | AG(25mg/kg) | 8 |  | 0.025 |
|  | AG(50mg/kg) | 8 |  | 0.0047 |
| Fig11I | Saline | 8 | One-way ANOVA followed by bonferroni post hoc tests | / |
|  | Morphine | 8 |  | 0.0001 |
|  | AG(25mg/kg) | 8 |  | 0.0001 |
|  | AG(50mg/kg) | 8 |  | 0.0001 |
| Fig11D | Saline | 8 | One-way ANOVA followed by bonferroni post hoc tests | / |
|  | Morphine | 8 |  | 0.0018 |
|  | AG(25mg/kg) | 8 |  | 0.027 |
|  | AG(50mg/kg) | 8 |  | 0.023 |
| FigS1A | SNI+saline | 8 | Two-way ANOVA followed by bonferroni post hoc tests | / |
|  | SNI+morphine | 8 |  | 0.0001 |
|  | SNI+AG(25mg/kg) | 8 |  | 0.0073 |
|  | SNI+AG(50mg/kg) | 8 |  | 0.0001 |
| FigS2B | Formalin+saline | 7 | One-way ANOVA followed by bonferroni post hoc tests | / |
|  | Formalin+AG(5mg/kg) | 8 |  | ns |
|  | Formalin+AG(10mg/kg) | 7 |  | 0.0056 |
|  | Formalin+AG(25mg/kg) | 8 |  | 0.0047 |
|  | Formalin+AG(50mg/kg) | 10 |  | 0.0016 |
|  | Formalin+morphine | 9 |  | 0.0071 |
| FigS2C | Formalin+saline | 10 | One-way ANOVA followed by bonferroni post hoc tests | / |
|  | Formalin+AG(5mg/kg) | 10 |  | ns |
|  | Formalin+AG(10mg/kg) | 10 |  | 0.0094 |
|  | Formalin+AG(25mg/kg) | 8 |  | 0.038 |
|  | Formalin+AG(50mg/kg) | 9 |  | 0.0083 |
|  | Formalin+morphine | 10 |  | 0.0012 |
| FigS3B | CFA+saline | 6 | Two-way ANOVA followed by bonferroni post hoc tests | / |
|  | CFA+AG(5mg/kg) | 6 |  | 0.015 |
|  | CFA+AG(10mg/kg) | 6 |  | 0.0058 |
|  | CFA+AG(25mg/kg) | 6 |  | 0.0001 |
|  | CFA+AG(50mg/kg) | 6 |  | 0.0001 |
|  | CFA+morphine | 6 |  | 0.0051 |
| FigS3D | CFA+saline | 6 | Two-way ANOVA followed by bonferroni post hoc tests | / |
|  | CFA+AG(5mg/kg) | 6 |  | 0.027 |
|  | CFA+AG(25mg/kg) | 6 |  | 0.0084 |
|  | CFA+AG(50mg/kg) | 6 |  | 0.0001 |
|  | CFA+morphine | 6 |  | 0.0039 |
| FigS4B | Capsaicin+saline | 8 | Two-way ANOVA followed by bonferroni post hoc tests | / |
|  | Capsaicin+AG(10mg/kg) | 8 |  | 0.039 |
|  | Capsaicin+AG(25mg/kg) | 8 |  | 0.0068 |
|  | Capsaicin+AG(50mg/kg) | 8 |  | 0.0057 |
|  | Capsaicin+morphine | 8 |  | 0.018 |
| FigS5B | Acetic acid+saline | 8 | One-way ANOVA followed by bonferroni post hoc tests | / |
|  | Acetic acid+AG(5mg/kg) | 8 |  | ns |
|  | Acetic acid+AG(10mg/kg) | 8 |  | 0.023 |
|  | Acetic acid+AG(25mg/kg) | 8 |  | 0.0068 |
|  | Acetic acid+AG(50mg/kg) | 8 |  | 0.0074 |
|  | Acetic acid+morphine | 8 |  | 0.0037 |
